# Association between endometriosis and adverse reproductive and perinatal outcomes in women undergoing assisted reproductive technology: a systematic review and meta-analysis

**DOI:** 10.3389/fmed.2026.1630529

**Published:** 2026-01-28

**Authors:** Yan Wei, Xiong Xiao, Xixi Wu, Cong Xie, Tingting Li

**Affiliations:** Department of Gynecology, Chengdu Women’s and Children’s Central Hospital, School of Medicine, University of Electronic Science and Technology of China, Chengdu, China

**Keywords:** endometriosis, assisted reproductive technology, adverse pregnancy outcomes, preterm birth, placenta previa, meta-analysis

## Abstract

**Background:**

With the increasing use of assisted reproductive technology (ART), more women with endometriosis are achieving pregnancy through ART. However, the impact of endometriosis on pregnancy and perinatal outcomes following ART remains controversial. This study aimed to clarify the association between endometriosis and adverse pregnancy and perinatal outcomes through a meta-analysis.

**Methods:**

A systematic search of PubMed, Embase, Web of Science, and Cochrane Library was conducted to identify relevant studies published before March 12, 2025. Cohort studies comparing adverse pregnancy and perinatal outcomes between women with and without endometriosis undergoing ART were included. Meta-analysis was performed using STATA 12.0 and R 4.3.2 software to calculate risk ratios (RRs) and 95% confidence intervals (CIs) for the association between endometriosis and adverse outcomes. Heterogeneity among studies was quantified using Cochran’s Q test, I^2^ statistics, and 95% prediction intervals (PIs). Subgroup analyses, sensitivity analyses, and publication bias assessments were also conducted.

**Results:**

A total of 29 cohort studies, including 93,071 women with endometriosis and 1,350,005 controls, were included in the meta-analysis. Compared with women without endometriosis, those with endometriosis undergoing ART had significantly lower clinical pregnancy rate (RR 0.850, 95% CI 0.726–0.994, 95% PI 0.570–1.267) and live birth rate (RR 0.716, 95% CI 0.556–0.923, 95% PI 0.341–1.504). They were also at higher risk for preterm birth (RR 1.277, 95% CI 1.187–1.373, 95% PI 1.024–1.591), placenta previa (RR 2.246, 95% CI 1.759–2.869, 95% PI 1.028–4.910), postpartum hemorrhage (RR 1.310, 95% CI 1.198–1.432, 95% PI 1.154–1.486), cesarean section (RR 1.296, 95% CI 1.165–1.441, 95% PI 0.944–1.779), low birth weight (RR 1.159, 95% CI 1.050–1.279, 95% PI 1.025–1.310), stillbirth (RR 1.219, 95% CI 1.032–1.440, 95% PI 0.930–1.597), and hypertensive disorders of pregnancy (RR 1.161, 95% CI 1.096–1.231, 95% PI 1.057–1.276). However, no significant differences were observed between the two groups in the risk of small for gestational age, miscarriage, preeclampsia, large for gestational age, or ectopic pregnancy (all *p* > 0.05). Subgroup analyses revealed variations in outcomes based on ethnicity, endometriosis stage, and mode of ART, but the overall results were robust.

**Conclusion:**

Endometriosis significantly impacts pregnancy and perinatal outcomes following ART, increasing the risk of multiple adverse outcomes. These findings provide critical evidence for individualized reproductive treatment and perinatal care in women with endometriosis.

## 1 Introduction

Endometriosis is a common chronic gynecological disorder characterized by the presence of functional endometrial-like tissue outside the uterine cavity, often accompanied by chronic inflammation and fibrotic changes (1). The prevalence of endometriosis is estimated to be approximately 10% among women of reproductive age, and it significantly impacts female fertility, being recognized as one of the leading causes of infertility in women (2). Studies have shown that women with endometriosis have substantially lower natural conception rates compared to those without the condition, particularly in cases of moderate to severe disease (3, 4). With the widespread application of assisted reproductive technology (ART), including *in vitro* fertilization (IVF) and intracytoplasmic sperm injection (ICSI), an increasing number of women with endometriosis are achieving pregnancy through these methods. However, existing evidence suggests that endometriosis may not only reduce the success rates of ART but also increase the risk of adverse pregnancy and perinatal outcomes (5, 6).

Previous research has demonstrated a strong association between endometriosis and various adverse pregnancy and perinatal outcomes. For instance, women with endometriosis were at a higher risk of preterm birth, which may be linked to chronic inflammation and alterations in the endometrial environment caused by the disease (7). Some studies have reported that women with endometriosis experience lower clinical pregnancy and live birth rates, as well as an increased risk of complications such as preterm birth, low birth weight (LBW), and hypertensive disorders of pregnancy (HDP) (8–10). Additionally, endometriosis has been associated with placental abnormalities, including placental abruption, abnormal placental implantation, and placenta previa, likely due to uterine damage and impaired endometrial function (11, 12). Furthermore, women with endometriosis undergoing ART were reported to have higher rates of cesarean section, placenta previa, and ectopic pregnancy compared to those without the condition (13–15). However, studies investigating these adverse outcomes showed considerable heterogeneity, which may be attributed to differences in sample sizes, endometriosis stage, mode of ART, and the extent of adjustment for confounding factors. This heterogeneity limited the comparability and generalizability of the findings.

Although systematic reviews and meta-analyses have previously explored the relationship between endometriosis and adverse pregnancy outcomes (16–18), these studies were published some time ago and often did not focus specifically on women with endometriosis undergoing ART. Moreover, current research has insufficiently addressed subgroup analyses related to disease stage, ethnic differences, and ART modalities. Considering the increasing prevalence of ART procedures among women diagnosed with endometriosis, it becomes crucial to explore the influence of this condition on ART outcomes. Gaining a deeper understanding of this relationship could offer essential guidance for tailoring reproductive advice and enhancing therapeutic approaches to meet the specific needs of these patients. Therefore, this study aimed to perform a meta-analysis to evaluate the risk of adverse pregnancy and perinatal outcomes in women with endometriosis undergoing ART. Additionally, this study seeks to investigate the potential impact of subgroup factors, including race, disease stage, and ART type, on the observed associations.

## 2 Methods

### 2.1 Protocol registration

This study followed the principles outlined in the Preferred Reporting Items for Systematic Reviews and Meta-Analyses (PRISMA) framework (19). Additionally, the research protocol was registered in the PROSPERO database with the identifier CRD420251021677.

### 2.2 Search strategy

A systematic search of multiple electronic databases, including PubMed, Embase, Web of Science, and the Cochrane Library, was conducted to identify studies published from their inception up to March 12, 2025, investigating the association between endometriosis and reproductive or perinatal outcomes. The search strategy employed a combination of keywords, such as (“endometriosis” or “endometrioses” or “endometrioma” or “endometriomas”) AND (“pregnancy outcome” or “obstetric outcome” or “reproductive outcome” or “fertility outcome” or “preterm” or “cesarean section” or “small for gestational age” or “placenta previa” or “preeclampsia” or “postpartum hemorrhage” or “miscarriage” or “stillbirth” or “hypertensive disorders of pregnancy”) AND (“cohort study” or “cohort studies” or “longitudinal”). Supplementary Files 1 provided a detailed description of the search methodology applied to each database. No language restrictions were imposed during the search process. Furthermore, the reference lists of the relevant review articles included were manually reviewed to identify any additional relevant studies that were potentially missed in the database query.

### 2.3 Inclusion and exclusion criteria

For inclusion in this meta-analysis, studies were required to fulfill the following criteria: (i) Studies employed a cohort design to evaluate the relationship between endometriosis and adverse pregnancy outcomes; (ii) The exposure group consisted of women diagnosed with endometriosis who underwent ART; (iii) The control group comprised women without endometriosis who also underwent ART; (iv) The studies reported either risk ratios (RRs) or odds ratios (ORs) along with their 95% confidence intervals (CIs) for the association between endometriosis and adverse reproductive or perinatal outcomes. Exclusion criteria included: (i) Studies with a case–control or cross-sectional design; (ii) The reported outcomes were based on mixed populations of women who conceived either naturally or through ART; (iii) Studies that did not report required outcomes; (iv) Conference abstracts, reviews, case reports, editorials, or commentaries.

### 2.4 Data extraction and quality assessment

The screening of literature, extraction of data, and assessment of study quality were independently conducted by two researchers. Any discrepancies between the two were resolved through consultation with a third reviewer to reach a consensus. The extracted data encompassed the following categories: (i) study characteristics, including the first author’s name, publication year, study design, geographic location, and sample size; (ii) participant information, such as the diagnosis of endometriosis, its stage or phenotype, maternal age, method of conception, and whether surgical intervention for endometriosis had been performed; (iii) outcomes included in the meta-analysis and adjusted confounding variables. The quality of the included cohort studies was assessed using the Newcastle-Ottawa Scale (NOS) (20), which evaluates three dimensions: cohort selection (4 items, 0–4 stars), cohort comparability (1 item, 0–2 stars), and outcome assessment (3 items, 0–3 stars). Each study was assigned a total score ranging from 0 to 9, with studies scoring 0–4 considered low quality, those scoring 5–6 categorized as moderate quality, and scores of 7–9 reflecting high-quality studies (21).

### 2.5 Statistical analysis

Meta-analyses were performed using STATA software (version 12.0) and R software (version 4.3.2). To evaluate the relationship between endometriosis and adverse pregnancy outcomes, RRs accompanied by 95% CIs were calculated. The degree of statistical heterogeneity across the included studies was examined using Cochran’s Q test, alongside the I^2^ and Tau^2^ statistics, as well as the 95% prediction interval (PI) (22, 23). Criteria for significant heterogeneity included *p* < 0.1 or I^2^ > 50%. When heterogeneity was detected, estimates were pooled using a random-effects model; otherwise, a fixed-effects model was applied (24). Subgroup analyses were conducted for groups with at least two included studies to analyze the effects of ethnicity, endometriosis stage, and categories of ART on the outcomes. Sensitivity analyses were conducted to assess the robustness of the results by excluding individual studies. Publication bias was evaluated using Begg and Mazumdar’s (25) and Egger’s et al. (26) tests. Statistical significance was defined as a two-sided *p*-value below 0.05.

## 3 Results

### 3.1 Study selection

**Figure 1:**
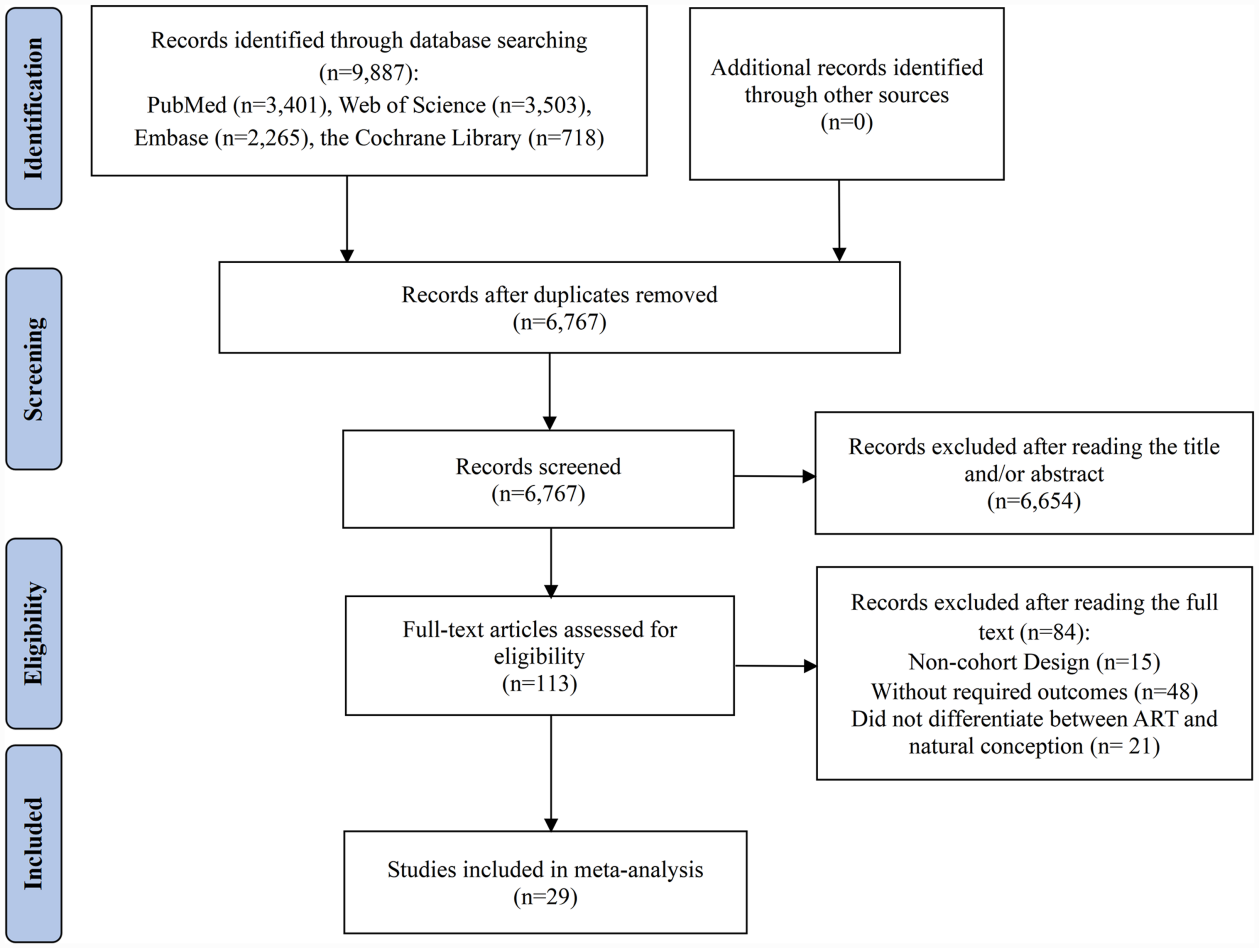
Flow diagram of the process of study selection.

A comprehensive search of the databases initially identified 9,887 articles for potential inclusion. After the removal of duplicate entries, 6,767 records remained. Titles and abstracts were then screened, resulting in the exclusion of 6,654 articles that were deemed unrelated to the research focus. Subsequently, 113 full-text articles underwent detailed evaluation to determine their eligibility. Following the application of inclusion and exclusion criteria, 84 studies were eliminated from consideration for the meta-analysis. Among these, 15 lacked a cohort design, 48 failed to provide essential RRs or ORs alongside 95% CIs for adverse pregnancy outcomes, and 21 did not distinguish between ART and natural conception in individuals with endometriosis. Ultimately, 29 studies met the eligibility criteria and were included in the meta-analysis (7, 9–11, 13–15, 27–48) (Figure 1).

### 3.2 Characteristics and quality assessment of the included studies

The principal characteristics of the included studies and their participant cohorts were presented in Table 1. To ensure the incorporation of up-to-date and pertinent findings, only studies published from 2010 onward were considered for the meta-analysis. A total of 29 studies were ultimately included, encompassing 93,071 individuals diagnosed with endometriosis and 1,350,005 controls. Notably, two studies did not specify the number of participants analyzed. The primary outcomes assessed in the meta-analysis included clinical pregnancy rate, live birth rate, preterm birth, small for gestational age (SGA), placenta previa, and miscarriage. Secondary outcomes comprised preeclampsia, postpartum hemorrhage, cesarean section, LBW, large for gestational age (LGA), ectopic pregnancy, stillbirth, and HDP. Preterm birth was defined as delivery occurring before 37 completed weeks of gestation, while SGA and LGA were categorized as birth weights below the 10th percentile or above the 90th percentile for gestational age, respectively (49). LBW was defined as a birth weight under 2,500 g. All cohort studies included in the analysis were evaluated as being of high quality, as they provided detailed descriptions of their study designs (Supplemental Files 2).

**Table 1:** Characteristics of the included studies investigating the association between endometriosis and adverse reproductive and perinatal outcomes in women undergoing assisted reproductive technology.

First author (year)	Study design	Country/region	Diagnosis of endometriosis	Endometriosis stage/phenotype	Sample size (E/C)	Maternal age (E/C, years)	Mode of conception	Surgically treated endometriosis	Outcomes in meta-analysis	Adjustments
Gebremedhin et al. (2024) (31)	RCS	Australia	ICD-AM codes	All stages	3,278/19,142	15–49	ART	NR	Preeclampsia, PP, and PTB	Maternal age, birth year, socio-economic status, parity, and ethnicity/race
Yang et al. (2019) (47)	RCS	China	Laparoscopy or laparotomy	All stages	1,006/2,012	33.04 ± 3.66/32.83 ± 3.76	IVF/ICSI	Surgically treated and untreated endometriosis	Miscarriage	BMI, gravidity, parity, male factor infertility, total dose of FSH administered and number of oocytes retrieved
Glavind et al. (2017) (32)	Birth cohort study	Denmark	Histology	All stages	193/1,614	All age groups	ART	Surgically treated and untreated endometriosis	Preeclampsia, PTB, SGA, PH, and CS	Maternal age, maternal prepregnant BMI, parity, ethnicity, and years of school
Yu et al. (2022) (10)	RCS	China	NR	All stages	NR	NR	IVF	NR	PTB, SGA, LBW, and LGA	NR
Muteshi et al. (2018) (15)	RCS	United Kingdom	Laparoscopy	All stages	531/737	Median (range): 35 (23–44)/36 (19–44)	ART	NR	PTB, miscarriage, LBR, CPR, and EP	None
Vendittelli et al. (2025) (14)	RCS	France	Questionnaire survey	All stages	543/13,382	NR	ART	Surgically treated and untreated endometriosis	Preeclampsia, PP, PTB, SGA, PH, and stillbirth	Maternal age, maternal prepregnancy BMI, lives alone, geographic origin, and tobacco use during the pregnancy
Salmanov et al. (2024) (13)	RCS	Ukraine	Laparoscopy or vaginal ultrasound	All stages	102/11,117	All age groups	IVF	NR	Preeclampsia, PTB, SGA, CS, and stillbirth	Maternal age, BMI, parity, and year of birth of the child
Healy et al. (2010) (35)	RCS	Australia	NR	NR	1,265/5,465	Median (range): 34 (20–45)	IVF/ICSI	NR	PP and PH	Age, year of birth, country of birth Asia and Middle East, marital status, parity, any miscarriages or terminations of pregnancy, vertex presentation, tertiary and private hospital status, pre-eclampsia, APH, PP, PA, premature rupture of membranes, induced labour, caesarean section
Farland et al. (2022) (11)	Register-based cohort study	United States	ICD-9 and ICD-10 codes	All stages	537/5,263	22–53/18–56	ART	NR	PTB, SGA, CS, LBW, and HDP	Maternal age, body mass index, race, education, plurality, and birth year
Breintoft et al. (2022) (7)	Birth cohort study	Denmark	ICD-8 and ICD-10 codes	All stages	247/2,102	All age groups	ART	Surgically treated and untreated endometriosis	PTB	Maternal age, BMI, parity, years of school, country of birth, smoking during pregnancy, alcohol during pregnancy, and year of birth
Queiroz Vaz et al. (2017) (39)	RCS	Brazil	Laparoscopy or MRI	Deep endometriosis	27/154	18–40	IVF	Surgically treated and surgically untreated endometriosis	Miscarriage and CPR	None
Benaglia et al. (2012) (28)	RCS	Italy and Spain	Ultrasound	Ovarian endometrioma	78/156	35.6 ± 3.5/36.1 ± 3.1	IVF/ICSI	Surgically treated and surgically untreated endometriosis	PTB, SGA, CS, LBW, and LBR	Smoking, PTB, basal follicle-stimulating hormone and previous IVF
Sharma et al. (2020) (42)	PCS	India	Laparoscopy	rASRM stage III/IV	294/358	18–40	IVF	Surgically treated and untreated endometriosis	Miscarriage, LBR, and CPR	Age, ovarian reserve, the number of mature oocyte and embryos
Rombauts et al. (2014) (40)	RCS	Australia	NR	NR	316/4,221	NR	IVF	NR	PP	Endometrial tickness, smoking, embryo transfer type, and cycle type
Wu et al. (2021) (46)	RCS	China	Transvaginal ultrasonography or MRI or abdominopelvic surgery	Ovarian endometrioma	862/862	32.73 ± 4.51/32.82 ± 4.82	IVF/ICSI	Surgically treated and surgically untreated endometriosis	LBR	NR
Sunkara et al. (2021) (43)	Register-based cohort study	United Kingdom	NR	All stages	5,053/112,300	≥ 18	IVF/ICSI	NR	PTB and LBW	Female age category, year of treatment, previous live birth, IVF or ICSI, number of embryos transferred and fresh or frozen cycles
Hjordt Hansen et al. (2014) (36)	Register-based cohort study	Denmark	ICD-8 and ICD-10 codes	All stages	NR	NR	ART	NR	Miscarriage and EP	None
Carusi et al. (2022) (29)	RCS	United States	ICD codes	All stages	1,259/21,086	All age groups	ART	NR	PP	Advanced maternal age, history of previous cesarean delivery, and multiple gestations
González-Comadran et al. (2017) (34)	RCS	Latin America	NR	All stages	3,583/18,833	34.83 ± 3.47/34.61 ± 3.91	IVF/ICSI	NR	Miscarriage, LBR, and CPR	Age of the female partner and number of embryos transferred
Fujii et al. (2016) (30)	RCS	Japan	Laparoscopy	All stages	92/512	Mean: 34.7/35.4	ART	Surgically treated and untreated endometriosis	PP, PTB, and SGA	Age, parity, and the number of transferred embryos
Sharma et al. (2019) (41)	RCS	India	Laparoscopy	rASRM stage III/IV	355/466	32.67 ± 2.53/33.02 ± 3.4	IVF/ICSI	Surgically treated and untreated endometriosis	Miscarriage, LBR, and CPR	None
Zhang et al. (2024) (48)	Register-based cohort study	Australia	ICD-10-AM	Deep, ovarian, superficial, or other endometriosis	1,379/21,795	34.3 ± 4.0/34.9 ± 4.4	ART	NR	PTB, SGA, and LGA	Mother’s age, whether mother had previous pregnancy, type of treatment, whether intracytoplasmic sperm injection was performed, number of embryo(s) transferred, stage of embryo(s) transferred, ovarian hyperstimulation syndrome during cycle, gestational hypertension, maternal smoking during pregnancy, socioeconomic index for area quintile, year of birth, and gestational diabetes
Volodarsky-Perel et al. (2022) (44)	RCS	Canada	Surgery or ultrasound	rASRM stage III-IV	75/982	35.7 ± 3.9/35.6 ± 4.4	IVF	NR	PP and PTB	Female age at embryo transfer, body mass index, number of previous pregnancies, hypothyroidism, chronic hypertension, pregestational diabetes mellitus, antral follicle count, endometrial thickness on the oocyte maturation trigger day, ICSI cycles, embryo stage at transfer, frozen embryo transfer cycles, number of transferred embryos, pre-eclampsia, GDM, newborn gender, PTB, and birthweight
Perkins et al. (2015) (38)	Register-based cohort study	United States	NR	All stages	51,086/327,937	NR	ART	NR	EP	Patient age, number of prior ART cycles, number of prior spontaneous abortions, number of prior live births, infertility diagnosis, year of ART procedure, use of assisted hatching, use of intracytoplasmic sperm injection, day of embryo transfer, number of embryos transferred, number of supernumerary embryos cryopreserved, and ovarian hyperstimulation syndrome
Wu et al. (2020) (45)	RCS	China	Histology	NR	1,111/5,975	31.96 ± 3.50/31.74 ± 4.10	IVF/ICSI	NR	PTB, SGA, LBW, and LGA	Maternal age, duration of infertility, parity, history of preterm birth, antral follicle count, fertilization method, endometrial preparation, embryo stage, number of embryos transferred, endometrium thickness, mode of delivery and pregnancy-related complications
Alson et al. (2024) (27)	PCS	Sweden	Ultrasound	Deep-infiltrating endometriosis and/or endometrioma	234/806	32.3 ± 4.0/31.9 ± 4.0	IVF/ICSI	NR	LBR and CPR	Age, serum antimullerian hormone, BMI, protocol, stimulation days, follicle-stimulating hormone dose, and day for embryo transfer
Gómez-Pereira et al. (2023) (33)	RCS	Spain	Laparoscopy or vaginal ultrasound	All stages	45/1,125	NR	IVF	Surgically treated and untreated endometriosis	PP	None
Lee et al. (2022) (37)	RCS	China	Laparoscopy or ultrasound	All stages	421/3,253	NR	IVF/ICSI	NR	HDP	None
Velez et al. (2022) (9)	RCS	Canada	ICD-9 codes	All stages	19,099/768,350	32.95 ± 4.88/30.03 ± 5.60	ART	Surgically treated and untreated endometriosis	PP, PTB, PH, CS, stillbirth, and HDP	Maternal age, income quantile, and history of fibroids

E, exposure group; C, control group; RCS, retrospective cohort study; PCS, prospective cohort study; ICD, International Classifcation of Diseases; AM, Australian Modifcation; ART, assisted reproductive technology; NR, not reported; IVF, in vitro fertilization; ICSI, intracytoplasmic sperm injection; MRI, magnetic resonance imaging; rASRM, revised American Society for Reproductive Medicine classification; ICD-10-AM, International Statistical Classification of Diseases and Related Health Problems, 10th revision, Australian Modification; CPR, clinical pregnancy rate; LBR, live birth rate; PTB, preterm birth; SGA, small for gestational age; PP, placenta previa; PH, postpartum hemorrhage; CS, cesarean section; LBW, low birth weight; LGA, large for gestational age; EP, ectopic pregnancy; HDP, hypertensive disorders of pregnancy.

### 3.3 Pooled effect and subgroup analysis of the primary outcomes

#### 3.3.1 Clinical pregnancy rate

**Figure 2:**
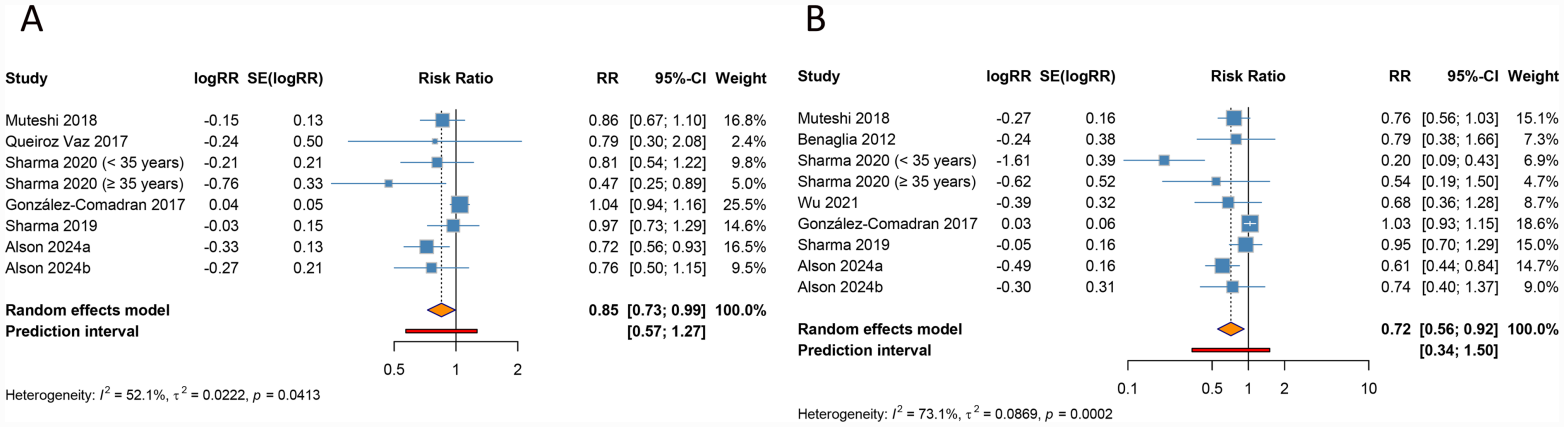
Forest plots of the association between endometriosis and clinical pregnancy rate **(A)** and live birth rate **(B)** in women undergoing assisted reproductive technology.

The meta-analysis identified a notable reduction in clinical pregnancy rate among women with endometriosis undergoing ART compared to those without endometriosis (RR 0.850, 95% CI 0.726–0.994, 95% PI 0.570–1.267), accompanied by significant heterogeneity across studies (I^2^ = 52.1%, Tau^2^ = 0.0222) (Table 2; Figure 2A). Ethnicity-based subgroup analysis indicated that this association was statistically significant among Caucasian populations (RR 0.784, 95% CI 0.666–0.923, 95% PI 0.548–1.122), whereas no significant relationship was observed in Asian (RR 0.786, 95% CI 0.554–1.115, 95% PI 0.231–2.673) or other ethnic groups (RR 1.041, 95% CI 0.936–1.157, 95% PI 0.522–2.074). Further stratification by endometriosis stage and ART method (IVF/ICSI) revealed no notable differences in all stages or within the IVF/ICSI subgroup (all *p* > 0.05) (Table 2; Supplementary Figure S1).

**Table 2:** Pooled effect and subgroup analysis of the association between endometriosis and primary adverse reproductive and perinatal outcomes.

Outcomes and subgroups	Number of studies	Meta-analysis				Heterogeneity	
		RR	95% CI	*p* value	95% PI	I^2^, Tau^2^	*p* value
Clinical pregnancy rate							
Overall	8	0.850	0.726–0.994	0.042	0.570–1.267	52.1%, 0.0222	0.041
Ethnicity							
Caucasian	3	0.784	0.666–0.923	0.004	0.548–1.122	0%, 0	0.610
Asian	3	0.786	0.554–1.115	0.177	0.231–2.673	51.7%, 0.0491	0.126
Others	2	1.041	0.936–1.157	0.465	0.522–2.074	0%, 0	0.576
Endometriosis stage							
All stages	2	1.013	0.918–1.117	0.802	0.182–5.282	49.5%, 0.0093	0.159
Others	6	0.784	0.673–0.914	0.002	0.639–0.962	0.4%, 0.0002	0.413
Mode of ART							
IVF/ICSI	7	0.838	0.693–1.012	0.066	0.513–1.368	57.3%, 0.0308	0.029
Live birth rate							
Overall	9	0.716	0.556–0.923	0.010	0.341–1.504	73.1%, 0.0869	<0.001
Ethnicity							
Caucasian	4	0.698	0.571–0.854	0.001	0.504–0.967	0%, 0	0.775
Asian	4	0.542	0.274–1.073	0.079	0.059–4.957	78.6%, 0.3622	0.003
Endometriosis stage							
All stages	2	0.917	0.685–1.228	0.560	0.046–18.242	70.9%, 0.0332	0.064
Others	7	0.633	0.455–0.880	0.007	0.258–1.549	60.0%, 0.1057	0.020
Mode of ART							
IVF/ICSI	8	0.698	0.518–0.941	0.018	0.294–1.659	75.1%, 0.1109	<0.001
Preterm birth							
Overall	21	1.277	1.187–1.373	<0.001	1.024–1.591	54.1%, 0.0098	0.002
Ethnicity							
Caucasian	7	1.289	1.208–1.375	<0.001	0.975–1.651	37.2%, 0.0078	0.145
Asian	5	1.626	1.024–2.583	0.039	0.394–6.716	81.6%, 0.2052	<0.001
Others	9	1.251	1.195–1.308	<0.001	1.089–1.432	27.9%, 0.0024	0.196
Endometriosis stage							
All stages	16	1.264	1.218–1.311	<0.001	1.185–1.344	6.5%, 0.0004	0.380
Others	5	1.522	0.744–3.115	0.250	0.158–14.628	85.1%, 0.5307	<0.001
Mode of ART							
IVF/ICSI	9	1.316	1.151–1.506	<0.001	0.920–1.884	73.1%, 0.0195	<0.001
Uncategorized	12	1.240	1.172–1.312	<0.001	1.088–1.424	14.5%, 0.0023	0.303
Small for gestational age							
Overall	14	0.994	0.915–1.080	0.891	0.743–1.285	28.0%, 0.0123	0.156
Ethnicity							
Caucasian	4	1.020	0.916–1.137	0.715	0.856–1.216	0%, 0	0.753
Asian	5	1.023	0.832–1.257	0.830	0.764–1.370	0%, 0	0.813
Others	5	0.955	0.693–1.316	0.778	0.362–2.516	71.6%, 0.0949	0.007
Endometriosis stage							
All stages	10	1.001	0.918–1.091	0.979	0.678–1.438	47.0%, 0.0221	0.049
Others	4	0.908	0.665–1.240	0.542	0.547–1.506	0%, 0	0.868
Mode of ART							
IVF/ICSI	6	1.025	0.924–1.139	0.639	0.894–1.176	0%, 0	0.926
Uncategorized	8	0.970	0.777–1.211	0.789	0.532–1.770	55.6%, 0.0518	0.027
Placenta previa							
Overall	11	2.246	1.759–2.869	<0.001	1.028–4.910	78.1%, 0.1076	<0.001
Ethnicity							
Caucasian	2	4.692	2.800–7.863	<0.001	0.165–133.404	0%, 0	0.418
Others	8	1.866	1.518–2.295	<0.001	1.017–3.424	70.9%, 0.0547	0.001
Endometriosis stage							
All stages	8	2.395	1.760–3.260	<0.001	0.929–6.175	84.0%, 0.1357	<0.001
Others	3	1.838	1.404–2.406	<0.001	1.018–3.320	0%, 0	0.425
Mode of ART							
IVF/ICSI	5	2.266	1.689–3.040	<0.001	1.058–4.852	54.5%, 0.0527	0.067
Uncategorized	6	2.277	1.537–3.375	<0.001	0.689–7.531	84.8%, 0.1762	<0.001
Miscarriage							
Overall	9	1.170	0.706–1.940	0.542	0.217–6.309	93.5%, 0.4673	<0.001
Ethnicity							
Caucasian	2	1.649	0.245–11.100	0.607	-	98.7%, 1.8687	<0.001
Asian	5	1.115	0.927–1.342	0.249	0.858–1.450	0%, 0	0.968
Others	2	1.042	0.862–1.260	0.669	0.305–3.567	0%, 0	0.500
Endometriosis stage							
All stages	5	1.305	0.687–2.480	0.417	0.146–11.662	96.7%, 0.5150	<0.001
Others	4	1.028	0.630–1.678	0.911	0.464–2.277	0%, 0	0.835
Mode of ART							
IVF/ICSI	7	1.079	0.945–1.232	0.261	0.914–1.273	0%, 0	0.974
Uncategorized	2	1.649	0.245–11.100	0.607	-	98.7%, 1.8687	<0.001

ART, assisted reproductive technology; IVF, in vitro fertilization; ICSI, intracytoplasmic sperm injection.

#### 3.3.2 Live birth rate

Live birth rate was found to be significantly lower in women with endometriosis undergoing ART relative to controls (RR 0.716, 95% CI 0.556–0.923, 95% PI 0.341–1.504), with significant heterogeneity present (I^2^ = 73.1%, Tau^2^ = 0.0869) (Table 2; Figure 2B). Subgroup analysis by ethnicity confirmed a significant reduction in live birth rate for Caucasian populations (RR 0.698, 95% CI 0.571–0.854, 95% PI 0.504–0.967), while findings for Asian groups were not statistically significant (RR 0.542, 95% CI 0.274–1.073, 95% PI 0.059–4.957). When examining endometriosis stage and ART method, a significant reduction in live birth rate was observed specifically within the IVF/ICSI subgroup (RR 0.698, 95% CI 0.518–0.941, 95% PI 0.294–1.659), while no differences were detected across all endometriosis stages (RR 0.917, 95% CI 0.685–1.228, 95% PI 0.046–18.242) (Table 2; Supplementary Figure S2).

#### 3.3.3 Preterm birth

**Figure 3:**
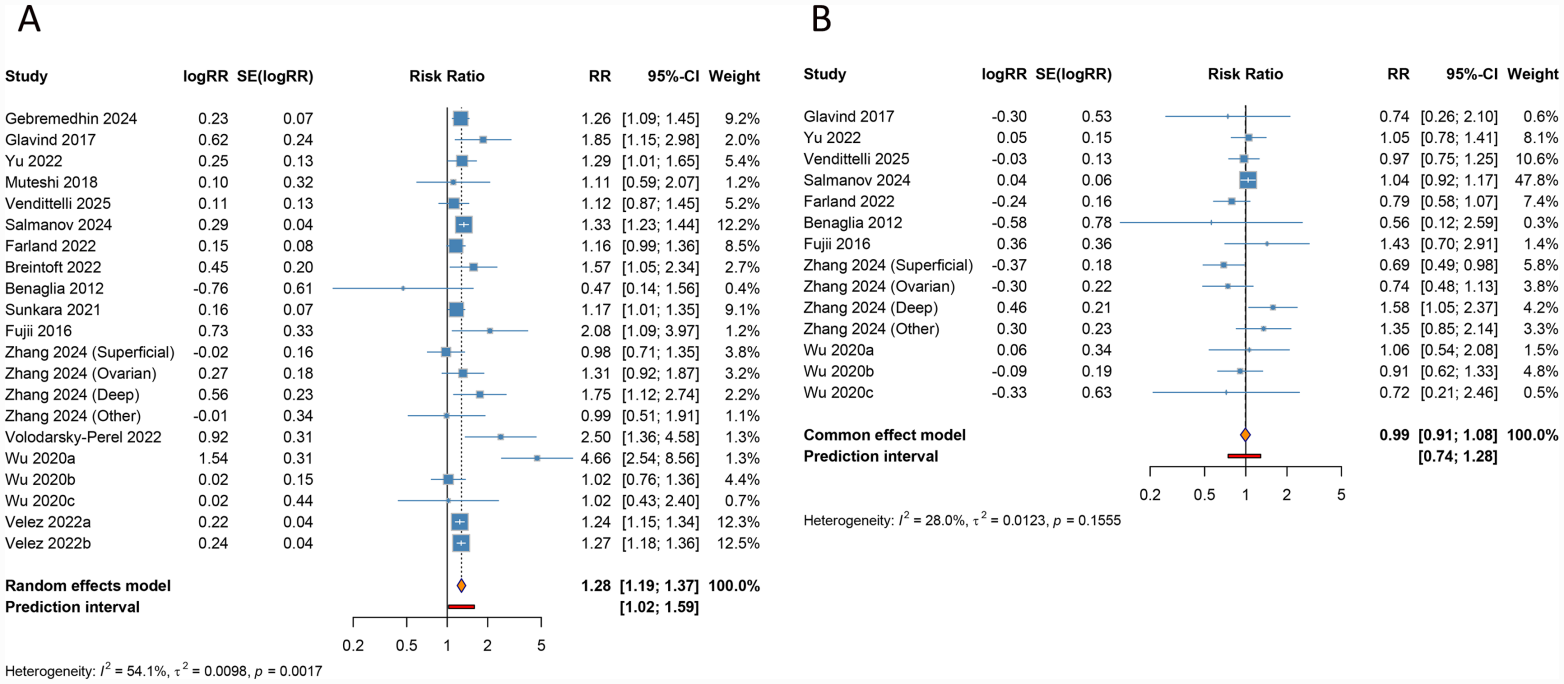
Forest plots of the association between endometriosis and preterm birth **(A)** and small for gestational age **(B)** in women undergoing assisted reproductive technology.

Endometriosis was found to be significantly associated with a higher risk of preterm birth (RR 1.277, 95% CI 1.187–1.373, 95% PI 1.024–1.591), with notable heterogeneity across studies (I^2^ = 54.1%, Tau^2^ = 0.0098) (Table 2; Figure 3A). Ethnicity-specific analysis demonstrated a consistent significant correlation in Caucasian (RR 1.289, 95% CI 1.208–1.375, 95% PI 0.975–1.651), Asian (RR 1.626, 95% CI 1.024–2.583, 95% PI 0.394–6.716), and other ethnic groups (RR 1.251, 95% CI 1.195–1.308, 95% PI 1.089–1.432). Stratification by disease stage indicated consistent risk across all stages of endometriosis (RR 1.264, 95% CI 1.218–1.311, 95% PI 1.185–1.344), and similar significant associations were observed irrespective of the ART modality employed (IVF/ICSI: RR 1.316, 95% CI 1.151–1.506, 95% PI 0.920–1.884; Uncategorized: RR 1.240, 95% CI 1.172–1.312, 95% PI 1.088–1.424) (Table 2; Supplementary Figure S3).

#### 3.3.4 Small for gestational age

The pooled data revealed no significant relationship between endometriosis and the risk of SGA (RR 0.994, 95% CI 0.915–1.080, 95% PI 0.743–1.285), with heterogeneity across studies remaining negligible (I^2^ = 28.0%, Tau^2^ = 0.0123) (Table 2; Figure 3B). Further subgroup analyses, stratified by ethnicity, endometriosis stage, and mode of ART, also failed to identify significant associations, suggesting that the presence of endometriosis does not substantially affect the likelihood of SGA in women undergoing ART (all *p* > 0.05) (Table 2; Supplementary Figure S4).

#### 3.3.5 Placenta previa

**Figure 4:**
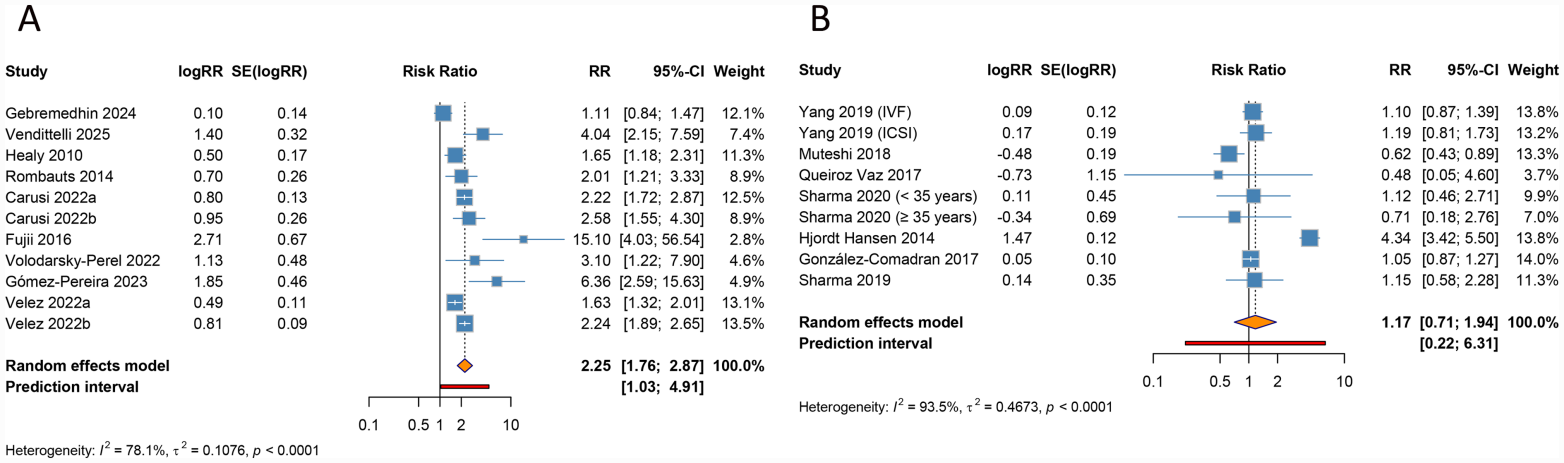
Forest plots of the association between endometriosis and placenta previa **(A)** and miscarriage **(B)** in women undergoing assisted reproductive technology.

Endometriosis was identified as a significant risk factor for placenta previa (RR 2.246, 95% CI 1.759–2.869, 95% PI 1.028–4.910), with high heterogeneity observed across studies (I^2^ = 78.1%, Tau^2^ = 0.1076) (Table 2; Figure 4A). Ethnicity-based subgroup analyses revealed notable associations in Caucasian populations (RR 4.692, 95% CI 2.800–7.863, 95% PI 0.165–133.404) as well as in other ethnic groups (RR 1.866, 95% CI 1.518–2.295, 95% PI 1.017–3.424). Further stratification by endometriosis stage revealed consistent associations within all stage subgroup (RR 2.395, 95% CI 1.760–3.260, 95% PI 0.929–6.175), while different ART approaches similarly showed significant correlations (IVF/ICSI: RR 2.266, 95% CI 1.689–3.040, 95% PI 1.058–4.852; Uncategorized: RR 2.277, 95% CI 1.537–3.375, 95% PI 0.689–7.531) (Table 2; Supplementary Figure S5).

#### 3.3.6 Miscarriage

The aggregated data indicated no significant connection between endometriosis and the risk of miscarriage (RR 1.170, 95% CI 0.706–1.940, 95% PI 0.217–6.309), with heterogeneity across studies remaining significant (I^2^ = 93.5%, Tau^2^ = 0.4673) (Table 2; Figure 4B). Subgroup analyses based on ethnicity, disease stage, and ART type also failed to reveal significant associations, suggesting that endometriosis does not substantially influence miscarriage risk in women undergoing ART (all *p* > 0.05) (Table 2; Supplementary Figure S6).

### 3.4 Pooled effect and subgroup analysis of the secondary outcomes

#### 3.4.1 Preeclampsia

**Figure 5:**
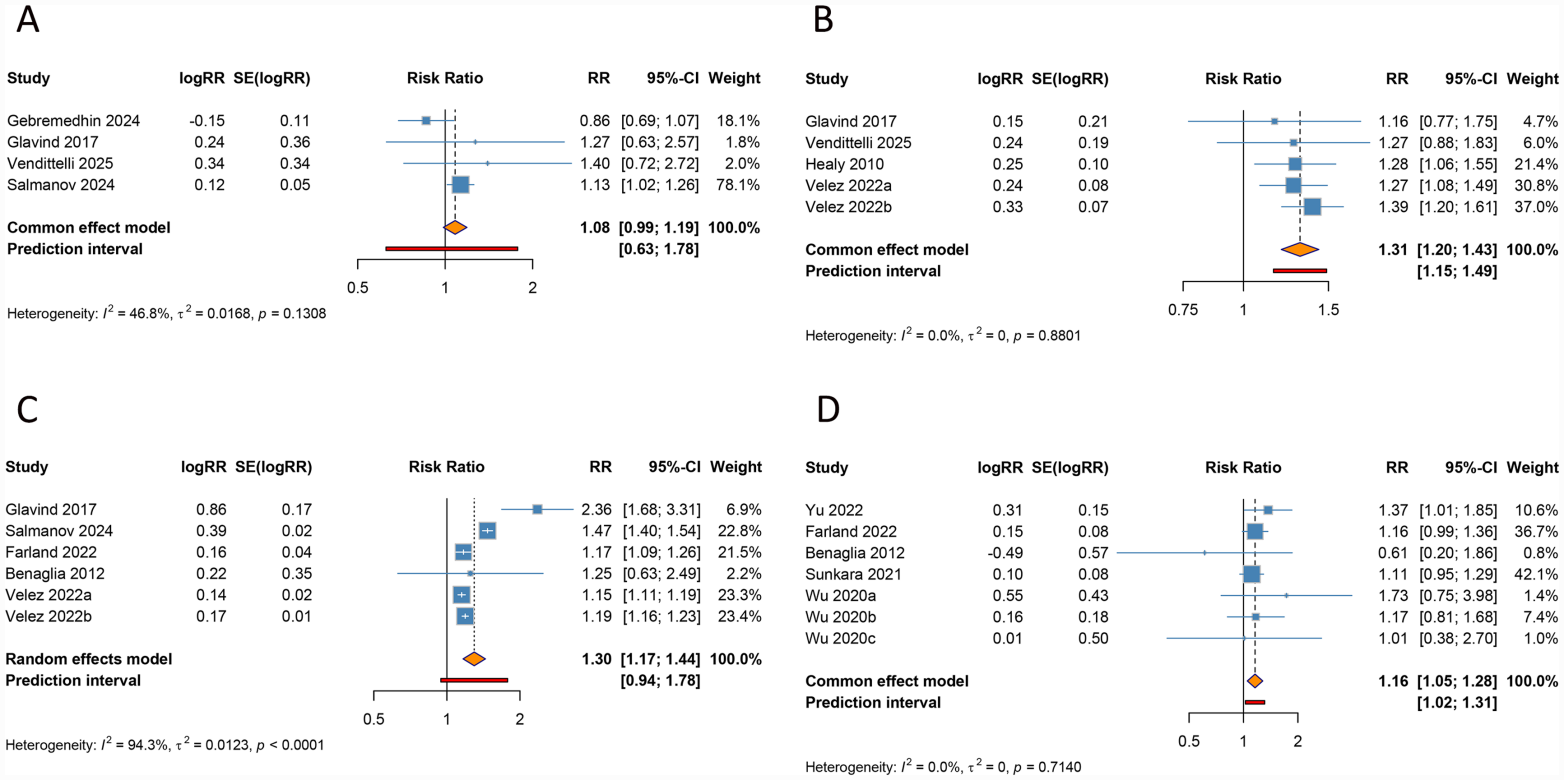
Forest plots of the association between endometriosis and preeclampsia **(A)**, postpartum hemorrhage **(B)**, cesarean section **(C)**, and low birth weight **(D)** in women undergoing assisted reproductive technology.

The meta-analysis revealed no significant correlation between endometriosis and preeclampsia (RR 1.082, 95% CI 0.986–1.188, 95% PI 0.626–1.777), with low heterogeneity noted across the studies included (I^2^ = 46.8%, Tau^2^ = 0.0168) (Table 3; Figure 5A). Subgroup analysis by ethnicity identified a significant association in Caucasian populations (RR 1.139, 95% CI 1.027–1.263, 95% PI 0.908–1.428), whereas no significant links were identified across other subgroups, including those categorized by endometriosis stage or ART modality (all *p* > 0.05) (Table 3; Supplementary Figure S7).

**Table 3:** Pooled effect and subgroup analysis of the association between endometriosis and secondary adverse reproductive and perinatal outcomes.

Outcomes and subgroups	Number of studies	Meta-analysis				Heterogeneity	
		RR	95% CI	*p* value	95% PI	I^2^, Tau^2^	*p* value
Preeclampsia							
Overall	4	1.082	0.986–1.188	0.097	0.626–1.777	46.8%, 0.0168	0.131
Ethnicity							
Caucasian	3	1.139	1.027–1.263	0.014	0.908–1.428	0%, 0	0.785
Endometriosis stage							
All stages	4	1.082	0.986–1.188	0.097	0.626–1.777	46.8%, 0.0168	0.131
Mode of ART							
Uncategorized	3	0.927	0.759–1.132	0.459	0.386–2.576	25.8%, 0.0242	0.260
Postpartum hemorrhage							
Overall	5	1.310	1.198–1.432	<0.001	1.154–1.486	0%, 0	0.880
Ethnicity							
Caucasian	2	1.220	0.930–1.602	0.151	0.209–7.120	0%, 0	0.746
Others	3	1.321	1.202–1.452	<0.001	1.073–1.626	0%, 0	0.673
Endometriosis stage							
All stages	4	1.318	1.192–1.458	<0.001	1.119–1.552	0%, 0	0.772
Mode of ART							
IVF/ICSI	2	1.349	1.200–1.516	<0.001	0.632–2.879	0%, 0	0.506
Uncategorized	3	1.257	1.094–1.444	0.001	0.927–1.704	0%, 0	0.920
Cesarean section							
Overall	6	1.296	1.165–1.441	<0.001	0.944–1.779	94.3%, 0.0123	<0.001
Ethnicity							
Caucasian	3	1.679	1.173–2.404	0.005	0.424–6.642	73.6%, 0.0686	0.023
Others	3	1.173	1.148–1.198	<0.001	1.108–1.241	8.1%, <0.0001	0.337
Endometriosis stage							
All stages	5	1.297	1.165–1.445	<0.001	0.918–1.832	95.5%, 0.0124	<0.001
Mode of ART							
IVF/ICSI	3	1.316	1.081–1.604	0.006	0.613–2.826	96.4%, 0.0214	<0.001
Uncategorized	3	1.275	1.101–1.477	0.001	0.718–2.263	88.3%, 0.0122	<0.001
Low birth weight							
Overall	7	1.159	1.050–1.279	0.003	1.025–1.310	0%, 0	0.714
Ethnicity							
Caucasian	2	1.098	0.945–1.276	0.223	0.105–11.019	8.0%, 0.0144	0.297
Asian	4	1.295	1.041–1.611	0.020	0.909–1.845	0%, 0	0.766
Endometriosis stage							
All stages	3	1.159	1.044–1.286	0.006	0.922–1.456	0%, 0	0.476
Others	4	1.158	0.856–1.567	0.342	0.708–1.893	0%, 0	0.524
Mode of ART							
IVF/ICSI	6	1.158	1.023–1.310	0.020	0.985–1.362	0%, 0	0.590
Large for gestational age							
Overall	8	0.942	0.781–1.135	0.530	0.591–1.501	50.1%, 0.0298	0.051
Ethnicity							
Asian	4	0.940	0.829–1.066	0.334	0.767–1.153	0%, 0	0.727
Others	4	0.809	0.462–1.416	0.457	0.137–4.776	74.8%, 0.2297	0.008
Endometriosis stage							
All stages	5	0.872	0.622–1.224	0.429	0.336–2.263	70.3%, 0.0881	0.009
Others	3	0.998	0.839–1.187	0.984	0.682–1.461	0%, 0	0.843
Mode of ART							
IVF/ICSI	4	0.940	0.829–1.066	0.334	0.767–1.153	0%, 0	0.727
Uncategorized	4	0.809	0.462–1.416	0.457	0.137–4.776	74.8%, 0.2297	0.008
Ectopic pregnancy							
Overall	3	1.973	0.789–4.931	0.146	0.053–73.205	84.1%, 0.4870	0.002
Ethnicity							
Caucasian	2	2.919	1.619–5.264	<0.001	0.064–133.440	0%, 0	0.439
Endometriosis stage							
All stages	3	1.973	0.789–4.931	0.146	0.053–73.205	84.1%, 0.4870	0.002
Mode of ART							
Uncategorized	3	1.973	0.789–4.931	0.146	0.053–73.205	84.1%, 0.4870	0.002
Stillbirth							
Overall	4	1.219	1.032–1.440	0.020	0.930–1.597	0%, 0	0.494
Ethnicity							
Caucasian	2	1.007	0.741–1.369	0.966	0.137–7.376	0%, 0	0.765
Others	2	1.320	1.083–1.608	0.006	0.366–4.760	0%, 0	0.652
Endometriosis stage							
All stages	4	1.219	1.032–1.440	0.020	0.930–1.597	0%, 0	0.494
Mode of ART							
IVF/ICSI	2	1.148	0.930–1.419	0.199	0.292–4.517	0%, 0	0.331
Uncategorized	2	1.344	1.026–1.760	0.032	0.233–7.734	0%, 0	0.422
Hypertensive disorders of pregnancy							
Overall	4	1.161	1.096–1.231	<0.001	1.057–1.276	0%, 0	0.819
Ethnicity							
Others	3	1.164	1.098–1.234	<0.001	1.024–1.323	0%, 0	0.911
Endometriosis stage							
All stages	4	1.161	1.096–1.231	<0.001	1.057–1.276	0%, 0	0.819
Mode of ART							
IVF/ICSI	2	1.174	1.079–1.277	<0.001	0.680–2.026	0%, 0	0.369
Uncategorized	2	1.150	1.062–1.246	0.001	0.685–1.930	0%, 0	0.999

ART, assisted reproductive technology; IVF, in vitro fertilization; ICSI, intracytoplasmic sperm injection.

#### 3.4.2 Postpartum hemorrhage

Endometriosis was significantly associated with an elevated risk of postpartum hemorrhage in the overall analysis (RR 1.310, 95% CI 1.198–1.432, 95% PI 1.154–1.486), with minimal heterogeneity detected (I^2^ = 0%, Tau^2^ = 0) (Table 3; Figure 5B). Subgroup analysis revealed a significant relationship in other ethnic groups (RR 1.321, 95% CI 1.202–1.452, 95% PI 1.073–1.626), whereas no such association was evident in Caucasian populations (RR 1.220, 95% CI 0.930–1.602, 95% PI 0.209–7.120). Notably, the elevated risk of postpartum hemorrhage remained consistent regardless of endometriosis stage (All stages: RR 1.318, 95% CI 1.192–1.458, 95% PI 1.119–1.552) and modes of ART (IVF/ICSI: RR 1.349, 95% CI 1.200–1.516, 95% PI 0.632–2.879; Uncategorized: RR 1.257, 95% CI 1.094–1.444, 95% PI 0.927–1.704) (Table 3; Supplementary Figure S8).

#### 3.4.3 Cesarean section

Endometriosis was significantly associated with an increased risk of cesarean section in the overall analysis (RR 1.296, 95% CI 1.165–1.441, 95% PI 0.944–1.779), accompanied by substantial heterogeneity (I^2^ = 94.3%, Tau^2^ = 0.0123) (Table 3; Figure 5C). Subgroup analyses based on ethnicity, disease stage, and ART modality demonstrated consistent associations, reinforcing the robustness of this finding (all *p* < 0.05) (Table 3; Supplementary Figure S9).

#### 3.4.4 Low birth weight

The meta-analysis identified a significant link between endometriosis and an increased risk of LBW (RR 1.159, 95% CI 1.050–1.279, 95% PI 1.025–1.310), with minimal heterogeneity across the included studies (I^2^ = 0%, Tau^2^ = 0) (Table 3; Figure 5D). Stratified analyses by ethnicity revealed that this relationship was significant among Asian populations (RR 1.295, 95% CI 1.041–1.611, 95% PI 0.909–1.845), whereas no evidence of association was observed in Caucasian cohorts (RR 1.098, 95% CI 0.945–1.276, 95% PI 0.105–11.019). Within all endometriosis stage and IVF/ICSI subgroups, the association remained consistent (all *p* < 0.05) (Table 3; Supplementary Figure S10).

#### 3.4.5 Large for gestational age

**Figure 6:**
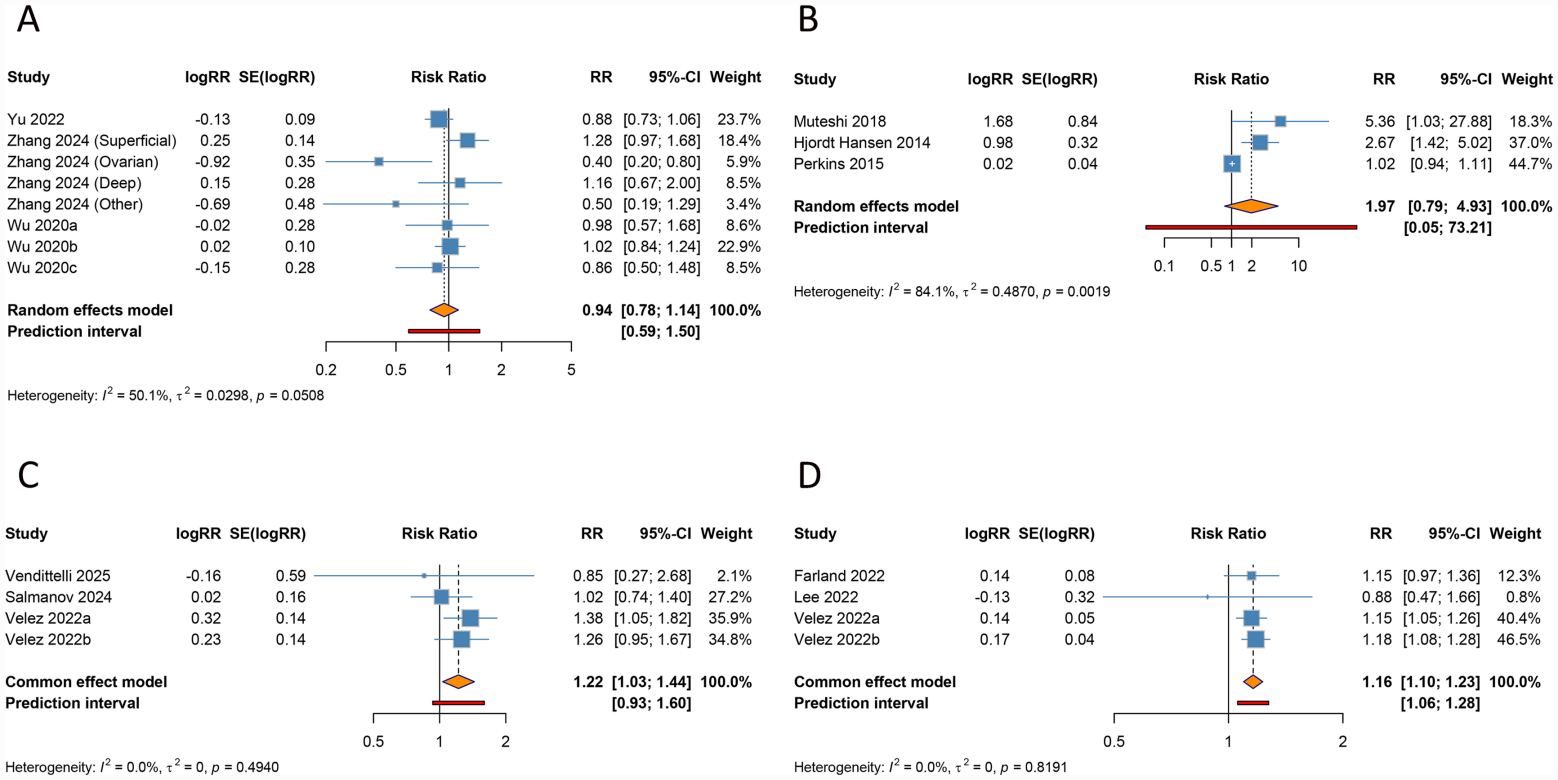
Forest plots of the association between endometriosis and large for gestational age **(A)**, ectopic pregnancy **(B)**, stillbirth **(C)**, and hypertensive disorders of pregnancy **(D)** in women undergoing assisted reproductive technology.

No significant association was observed between endometriosis and LGA in the overall analysis (RR 0.942, 95% CI 0.781–1.135, 95% PI 0.591–1.501), accompanied by moderate heterogeneity (I^2^ = 50.1%, Tau^2^ = 0.0298) (Table 3; Figure 6A). Subgroup analyses based on ethnicity, endometriosis stage, and mode of ART consistently failed to detect any significant relationships (all *p* > 0.05) (Table 3; Supplementary Figure S11).

#### 3.4.6 Ectopic pregnancy

The overall analysis suggested no significant association between endometriosis and ectopic pregnancy (RR 1.973, 95% CI 0.789–4.931, 95% PI 0.053–73.205), with significant heterogeneity (I^2^ = 84.1%, Tau^2^ = 0.4870) (Table 3; Figure 6B). Ethnicity-specific subgroup analysis uncovered a significant association between endometriosis and increased ectopic pregnancy risk within Caucasian populations (RR 2.919, 95% CI 1.619–5.264, 95% PI 0.064–133.440), while no significant links were observed across subgroups defined by endometriosis stage or ART modality (all *p* > 0.05) (Table 3; Supplementary Figure S12).

#### 3.4.7 Stillbirth

The overall analysis demonstrated a significant correlation between endometriosis and an elevated risk of stillbirth (RR 1.219, 95% CI 1.032–1.440, 95% PI 0.930–1.597), with negligible heterogeneity across studies (I^2^ = 0%, Tau^2^ = 0) (Table 3; Figure 6C). This association remained consistent among patients across all stages of endometriosis (RR 1.219, 95% CI 1.032–1.440, 95% PI 0.930–1.597). However, no significant relationship was identified in Caucasian populations or in subgroups undergoing IVF/ICSI (all *p* > 0.05) (Table 3; Supplementary Figure S13).

#### 3.4.8 Hypertensive disorders of pregnancy

Endometriosis was found to be significantly linked to a higher risk of HDP in the pooled analysis (RR 1.161, 95% CI 1.096–1.231, 95% PI 1.057–1.276), with minimal heterogeneity observed (I^2^ = 0%, Tau^2^ = 0) (Table 3; Figure 6D). Subgroup analyses stratified by ethnicity, endometriosis stage, and mode of ART consistently supported this association (all *p* < 0.05) (Table 3; Supplementary Figure S14).

### 3.5 Sensitivity analysis and publication bias

We performed sensitivity analysis and publication bias test for the pooled results of adverse pregnancy outcomes which included ≥ 10 studies. Sensitivity analysis involved recalculating pooled RRs and their associated 95% CIs following the systematic exclusion of individual studies, allowing an evaluation of the influence of each study on the aggregated results. This approach confirmed that the omission of any single study did not materially alter the conclusions, underscoring the reliability and stability of the findings (Supplementary Figure S15). Publication bias was assessed using Begg’s and Egger’s tests, both of which revealed no evidence of significant bias across the included studies (all *p* > 0.05). The funnel plots were provided in Supplementary Figure S16.

## 4 Discussion

This study conducted a meta-analysis of cohort studies to explore the impact of endometriosis on adverse pregnancy and perinatal outcomes in women undergoing ART. The results demonstrated that endometriosis significantly affects several critical pregnancy and perinatal outcomes, including clinical pregnancy rate, live birth rate, preterm birth, placenta previa, postpartum hemorrhage, cesarean section, LBW, stillbirth, and HDP. However, no statistically significant associations were observed for certain outcomes, such as SGA, miscarriage, preeclampsia, LGA, and ectopic pregnancy. Subgroup analyses based on ethnicity, endometriosis stage, and mode of ART further explored the associations between endometriosis and the aforementioned adverse pregnancy outcomes across different subpopulations.

Our findings revealed that women with endometriosis had significantly lower clinical pregnancy rate and live birth rate after ART compared to women without endometriosis. This result aligns with previous studies (27, 42), suggesting that endometriosis may impair ART success by affecting embryo implantation rates and pregnancy maintenance (50). The reduced endometrial receptivity in women with endometriosis is likely associated with chronic inflammation, local immune dysregulation, and endometrial fibrosis (51–53). These pathological changes may hinder embryo implantation and early pregnancy maintenance. Additionally, ovarian function in women with endometriosis may be compromised, leading to reduced oocyte quality and, consequently, diminished embryonic developmental potential (5). Our subgroup analyses indicated that the association of endometriosis and lower clinical pregnancy and live birth rate was more pronounced in Caucasian populations but less evident in Asian populations, indicating that racial differences may play a role in the influence of endometriosis on ART outcomes. These disparities could be attributed to variations in genetic background, disease phenotypes, and ART treatment strategies across populations.

In terms of perinatal outcomes, endometriosis significantly increased the risks of preterm birth, placenta previa, LBW, and stillbirth. Notably, the elevated risk of preterm birth was consistently observed across most subgroups, suggesting that endometriosis may influence pregnancy duration through multiple mechanisms. The endometrial environment in women with endometriosis may exhibit chronic inflammation, fibrosis, and abnormalities in angiogenesis (54), which could impair placental function and fetal growth. Additionally, endometriosis may alter the structure and function of the myometrium, increasing uterine contractility and thereby triggering preterm labor (55). The significantly increased risk of placenta previa may be related to the abnormal frequency and amplitude of uterine contractions observed in women affected (12). Previous studies have shown that reduced endometrial receptivity in these patients may lead to abnormal embryo implantation sites, thus increasing the likelihood of placenta previa (56). Furthermore, the heightened risk of LBW may be associated with placental insufficiency and intrauterine growth restriction (57), further supporting the adverse effects of endometriosis on placental development and function. Impaired placental perfusion in women with endometriosis could result in intrauterine growth restriction (58). Additionally, preterm birth is a major contributor to LBW, and the higher incidence of preterm birth in women with endometriosis may indirectly increase the risk of LBW. Moreover, the elevated risk of stillbirth in our study may reflect the cumulative adverse effects of endometriosis on pregnancy. Although the incidence of stillbirth is relatively low, its severity necessitates closer monitoring of high-risk patients in clinical practice.

This study also found that endometriosis was significantly associated with increased risks of HDP, cesarean section, and postpartum hemorrhage. The increased risk of HDP may be attributed to the chronic inflammatory state, endothelial dysfunction, and abnormal angiogenesis observed in women with endometriosis (59). These pathophysiological mechanisms could lead to inadequate placental perfusion and dysregulated maternal vascular tone, thereby increasing the risk of HDP (60). The elevated risk of postpartum hemorrhage may be related to uterine contractility disorders and abnormal placental implantation in women with endometriosis (61). Additionally, endometriosis may lead to myometrial dysfunction, increasing the risk of hemorrhage during delivery (62). Previous studies have suggested that endometriosis may cause myometrial fibrosis and vascular abnormalities (63), which could impair uterine contraction and placental separation during delivery. Furthermore, the significantly higher cesarean section rate may be due to the increased incidence of pregnancy complications (e.g., placenta previa, preterm birth) in women with endometriosis, prompting clinicians to opt for cesarean section to mitigate delivery-related risks, which in turn increases the risk of postpartum hemorrhage.

Although this study identified significant associations between endometriosis and several adverse perinatal outcomes, no statistically significant relationships were observed for certain outcomes, including SGA, miscarriage, preeclampsia, LGA, and ectopic pregnancy. These findings may reflect the selective impact of endometriosis on different pregnancy outcomes. For instance, the lack of a significant association between endometriosis and SGA suggested that while placental function may be impaired in women with endometriosis (64), it may not be sufficient to markedly increase the risk of intrauterine growth restriction. Moreover, the occurrence of SGA is influenced by multiple factors, including genetic predisposition, maternal nutritional status, and other pregnancy complications (65–67). Similarly, although endometriosis may increase the risk of miscarriage through inflammation, immune dysregulation, and implantation failure (3, 68), no significant association was observed in this study. This finding may be attributed to the rigorous selection of high-quality embryos during ART, which could reduce the likelihood of miscarriage. It is important to note, however, that the lack of a statistically significant association does not necessarily imply the absence of a true relationship. The relatively small number of studies reporting these results, along with limited sample sizes and racial differences among study populations, may have reduced the ability to detect statistically significant associations. Furthermore, variations in endometriosis staging and modes of conception may have contributed to residual heterogeneity, complicating the interpretation of these findings. Future studies with larger sample sizes and more homogeneous cohorts are needed to determine whether the observed lack of significant associations reflects a true absence of effect. Additionally, future research should further investigate the potential impact of different phenotypes or stages of endometriosis on these adverse pregnancy outcomes.

This study has some limitations. First, significant heterogeneity was observed among the included studies, which may be related to differences in participant characteristics, endometriosis stage, and mode of ART. Although subgroup analyses and random-effects models were applied to partially address heterogeneity, residual confounding factors (such as maternal age) cannot be entirely ruled out, as some studies adjusted for common confounding factors while others did not or failed to report such adjustments. Second, the control groups varied across studies, with some utilizing fertile individuals, others including subfertile patients, or those experiencing infertility due to male factors as non-endometriotic controls. Nevertheless, the majority of studies accounted for these differences by either adjusting for or restricting variables such as age, parity, or the number of pregnancies. Third, this study did not consider specific endometriosis phenotypes (e.g., deep infiltrating, ovarian, or superficial endometriosis) or adenomyosis comorbidity, which often coexists with endometriosis and shares adverse reproductive and obstetric outcomes. Adenomyosis has been shown to negatively impact fertility and pregnancy outcomes, including increased risks of hypertensive disorders, threatened preterm labor and postpartum maternal complications (69). Furthermore, the lack of standardized ART protocols across existing studies further limits the comparability of results. Future research should investigate these phenotypes and comorbidities to better understand their distinct impacts on ART outcomes. Fourth, due to the lack of detailed differentiation of ART methods in most studies, the current analysis is unable to compare outcomes between IVF and ICSI. Similarly, the majority of studies did not differentiate between the types of embryo transfer performed (e.g., frozen embryo transfer and fresh embryo transfer), which limits the feasibility of conducting further subgroup analyses. Future research should focus on comparing adverse pregnancy outcomes among various types of ART and different embryo transfer methods.

## 5 Conclusion

In summary, this study demonstrates that endometriosis significantly impacts pregnancy and perinatal outcomes following ART treatment. Specifically, endometriosis is associated with reduced clinical pregnancy and live birth rates, as well as an increased risk of preterm birth, placenta previa, postpartum hemorrhage, cesarean section, LBW, stillbirth, and HDP. Clinicians should implement closer surveillance for placenta previa and HDP, with early ultrasound screening and regular blood pressure monitoring. Multidisciplinary care is essential to manage risks such as preterm birth and LBW. Future research should further investigate the pathophysiological mechanisms of endometriosis and its effects on pregnancy outcomes, with the aim of optimizing treatment strategies and improving pregnancy management for affected patients.

## Data Availability

The original contributions presented in the study are included in the article/Supplementary material, further inquiries can be directed to the corresponding author.
