# Supplementary material for: Association between endometriosis and adverse reproductive and perinatal outcomes in women undergoing assisted reproductive technology: a systematic review and meta-analysis
Source: Front Med (Lausanne). 2026 Jan 28;13:1630529. doi: 10.3389/fmed.2026.1630529 (PMC12891222; doi:10.3389/fmed.2026.1630529)
Supplement: Supplementary file 1 [file Supplementary_file_1.docx]

**PubMed 3,401**

#1 (endometriosis) OR (endometrioses) OR (endometrioma) OR (endometriomas)

#2 (pregnancy outcome) OR (obstetric outcome) OR (reproductive outcome) OR (fertility outcome) OR (preterm) OR (premature labor) OR (premature delivery) OR (premature birth) OR (preterm labor) OR (preterm delivery) OR (preterm birth) OR (caesarean) OR (cesarean section) OR (c-section) OR (small for gestational age) OR (low birth weight) OR (placenta praevia) OR (placenta previa) OR (postpartum hemorrhage) OR (spontaneous hemoperitoneum) OR (antepartum hemorrhage) OR (pre-eclampsia) OR (preeclampsia) OR (gestational hypertension) OR (hypertensive disorders in pregnancy) OR (maternal hypertension) OR (pregnancy induced hypertension) OR (miscarriage) OR (stillbirth) OR (abruptio placentae) OR (placental abruption) OR (growth restriction) OR (gestational age) OR (livebirth rate) OR (fertilisation rate) OR (fertilization rate) OR (clinical pregnancy rate) OR (intestinal perforation) OR (bowel perforation) OR (cholestasis) OR (mid-trimester loss) OR (implantation rate) OR (ovarian response) OR (cycle cancellation)

#3 (cohort study) OR (cohort studies) OR (retrospective) OR (longitudinal) OR (follow-up) OR (prospective)

#4 #1 AND #2 AND #3

**Web of Science 3,503**

#1 TS=((endometriosis) OR (endometrioses) OR (endometrioma) OR (endometriomas))

#2 TS=((pregnancy outcome) OR (obstetric outcome) OR (reproductive outcome) OR (fertility outcome) OR (preterm) OR (premature labor) OR (premature delivery) OR (premature birth) OR (preterm labor) OR (preterm delivery) OR (preterm birth) OR (caesarean) OR (cesarean section) OR (c-section) OR (small for gestational age) OR (low birth weight) OR (placenta praevia) OR (placenta previa) OR (postpartum hemorrhage) OR (spontaneous hemoperitoneum) OR (antepartum hemorrhage) OR (pre-eclampsia) OR (preeclampsia) OR (gestational hypertension) OR (hypertensive disorders in pregnancy) OR (maternal hypertension) OR (pregnancy induced hypertension) OR (miscarriage) OR (stillbirth) OR (abruptio placentae) OR (placental abruption) OR (growth restriction) OR (gestational age) OR (livebirth rate) OR (fertilisation rate) OR (fertilization rate) OR (clinical pregnancy rate) OR (intestinal perforation) OR (bowel perforation) OR (cholestasis) OR (mid-trimester loss) OR (implantation rate) OR (ovarian response) OR (cycle cancellation))

#3 TS=((cohort study) OR (cohort studies) OR (retrospective) OR (longitudinal) OR (follow-up) OR (prospective))

#4 #1 AND #2 AND #3

**Embase 2,265**

#1 'endometriosis'/exp OR endometrioses OR 'endometrioma'/exp OR 'endometriomas'/exp

#2 'pregnancy outcome'/exp OR 'obstetric outcome'/exp OR 'reproductive outcome'/exp OR 'fertility outcome' OR preterm OR 'premature labor'/exp OR 'premature delivery'/exp OR 'premature birth'/exp OR 'preterm labor'/exp OR 'preterm delivery'/exp OR 'preterm birth'/exp OR caesarean OR 'cesarean section'/exp OR 'c section' OR 'small for gestational age'/exp OR 'low birth weight'/exp OR 'placenta praevia'/exp OR 'placenta previa'/exp OR 'postpartum hemorrhage'/exp OR 'spontaneous hemoperitoneum'/exp OR 'antepartum hemorrhage'/exp OR 'pre eclampsia'/exp OR 'preeclampsia'/exp OR 'gestational hypertension'/exp OR 'hypertensive disorders in pregnancy' OR 'maternal hypertension'/exp OR 'pregnancy induced hypertension'/exp OR 'miscarriage'/exp OR 'stillbirth'/exp OR 'abruptio placentae'/exp OR 'placental abruption'/exp OR 'growth restriction' OR 'gestational age'/exp OR 'livebirth rate' OR 'fertilisation rate' OR 'fertilization rate'/exp OR 'clinical pregnancy rate'/exp OR 'intestinal perforation'/exp OR 'bowel perforation'/exp OR 'cholestasis'/exp OR 'mid-trimester loss' OR 'implantation rate'/exp OR 'ovarian response'/exp OR 'cycle cancellation'

#3 'cohort study'/exp OR 'cohort studies'/exp OR retrospective OR 'longitudinal'/exp OR 'follow up'/exp OR prospective

#4 #1 AND #2 AND #3

**The Cochrane Library 718**

#1 All Text=((endometriosis) OR (endometrioses) OR (endometrioma) OR (endometriomas))

#2 All Text=((pregnancy outcome) OR (obstetric outcome) OR (reproductive outcome) OR (fertility outcome) OR (preterm) OR (premature labor) OR (premature delivery) OR (premature birth) OR (preterm labor) OR (preterm delivery) OR (preterm birth) OR (caesarean) OR (cesarean section) OR (c-section) OR (small for gestational age) OR (low birth weight) OR (placenta praevia) OR (placenta previa) OR (postpartum hemorrhage) OR (spontaneous hemoperitoneum) OR (antepartum hemorrhage) OR (pre-eclampsia) OR (preeclampsia) OR (gestational hypertension) OR (hypertensive disorders in pregnancy) OR (maternal hypertension) OR (pregnancy induced hypertension) OR (miscarriage) OR (stillbirth) OR (abruptio placentae) OR (placental abruption) OR (growth restriction) OR (gestational age) OR (livebirth rate) OR (fertilisation rate) OR (fertilization rate) OR (clinical pregnancy rate) OR (intestinal perforation) OR (bowel perforation) OR (cholestasis) OR (mid-trimester loss) OR (implantation rate) OR (ovarian response) OR (cycle cancellation))

#3 All Text=((cohort study) OR (cohort studies) OR (retrospective) OR (longitudinal) OR (follow-up) OR (prospective))

#4 #1 AND #2 AND #3
