# Supplementary material for: Association between endometriosis and adverse reproductive and perinatal outcomes in women undergoing assisted reproductive technology: a systematic review and meta-analysis
Source: Front Med (Lausanne). 2026 Jan 28;13:1630529. doi: 10.3389/fmed.2026.1630529 (PMC12891222; doi:10.3389/fmed.2026.1630529)

**FIGURE S1** Subgroup analysis of clinical pregnancy rate. (A) Ethnicity = Caucasian; (B) Ethnicity = Asian; (C) Ethnicity = Others; (D) Endometriosis stage = All stages; (E) Endometriosis stage = Others; (F) Mode of ART = IVF/ICSI.


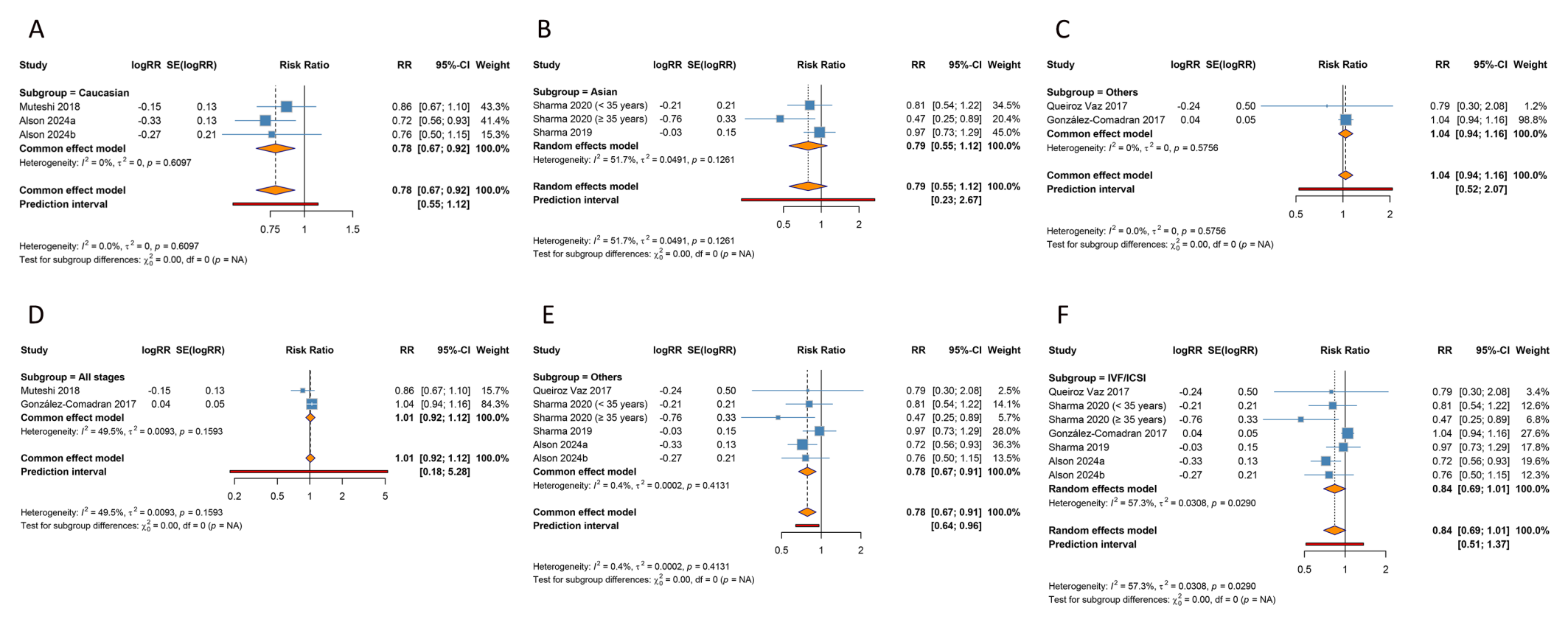


**FIGURE S2** Subgroup analysis of live birth rate. (A) Ethnicity = Caucasian; (B) Ethnicity = Asian; (C) Endometriosis stage = All stages; (D) Endometriosis stage = Others; (E) Mode of ART = IVF/ICSI.


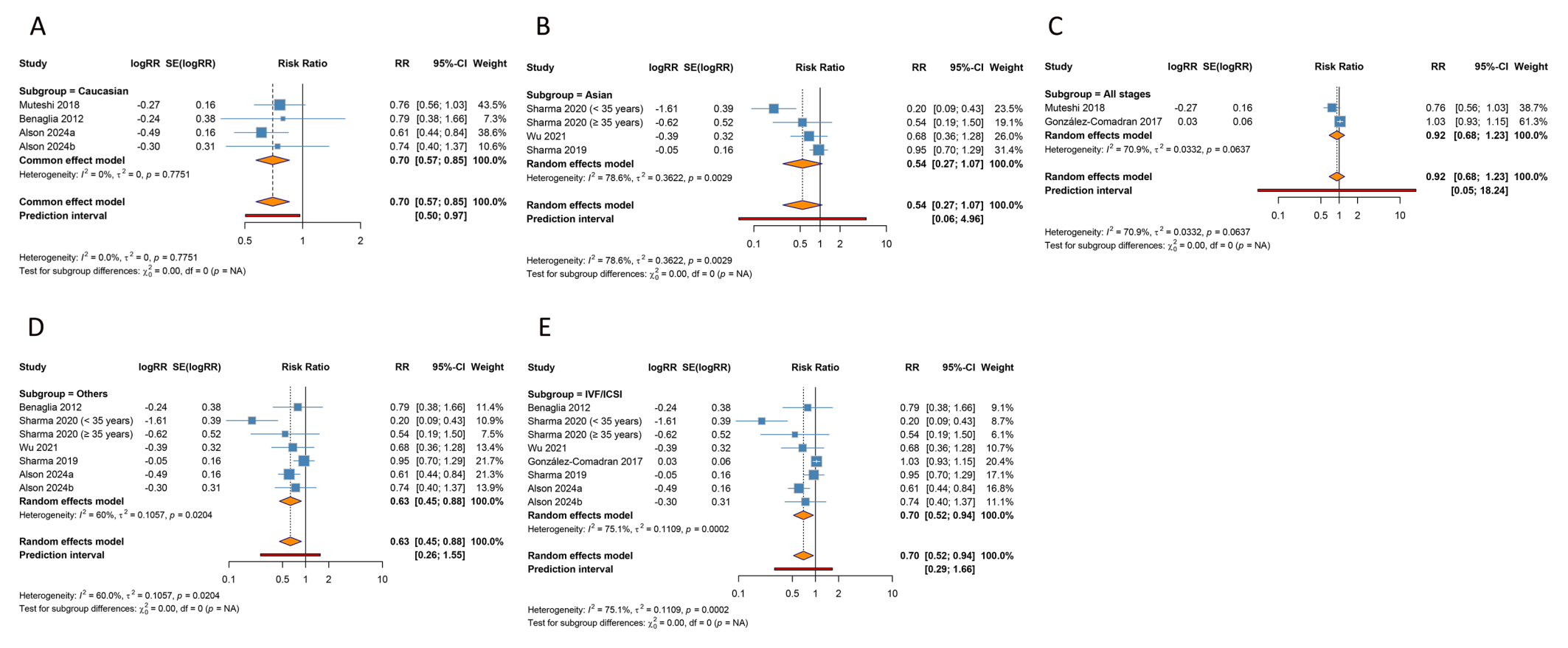


**FIGURE S3** Subgroup analysis of preterm birth. (A) Ethnicity = Caucasian; (B) Ethnicity = Asian; (C) Ethnicity = Others; (D) Endometriosis stage = All stages; (E) Endometriosis stage = Others; (F) Mode of ART = IVF/ICSI; (G) Mode of ART = Uncategorized.


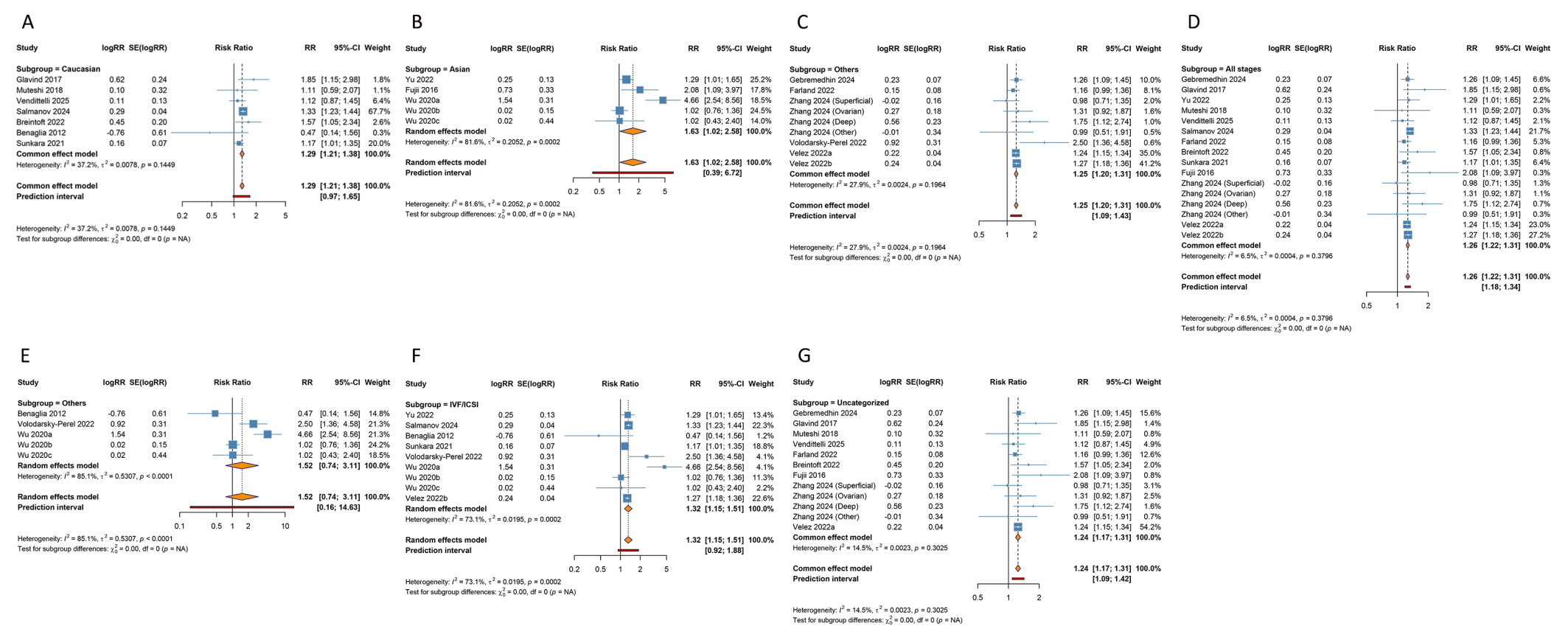


**FIGURE S4** Subgroup analysis of small for gestational age. (A) Ethnicity = Caucasian; (B) Ethnicity = Asian; (C) Ethnicity = Others; (D) Endometriosis stage = All stages; (E) Endometriosis stage = Others; (F) Mode of ART = IVF/ICSI; (G) Mode of ART = Uncategorized.


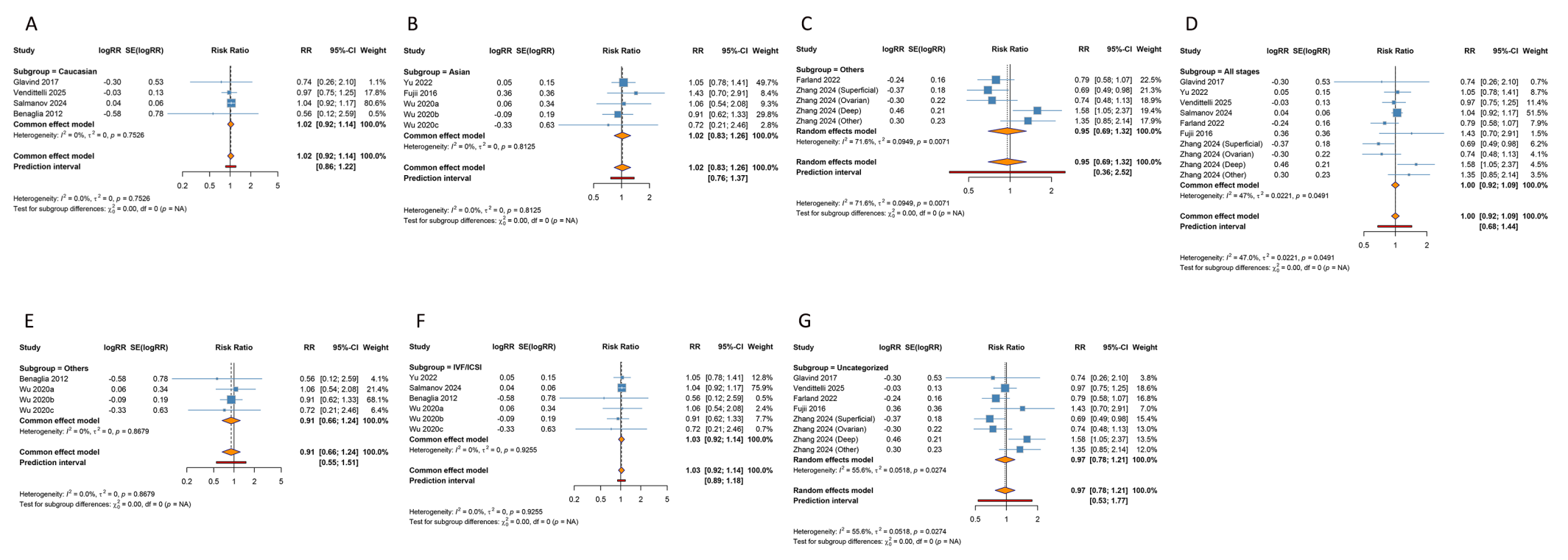


**FIGURE S5** Subgroup analysis of placenta previa. (A) Ethnicity = Caucasian; (B) Ethnicity = Others; (C) Endometriosis stage = All stages; (D) Endometriosis stage = Others; (E) Mode of ART = IVF/ICSI; (F) Mode of ART = Uncategorized.


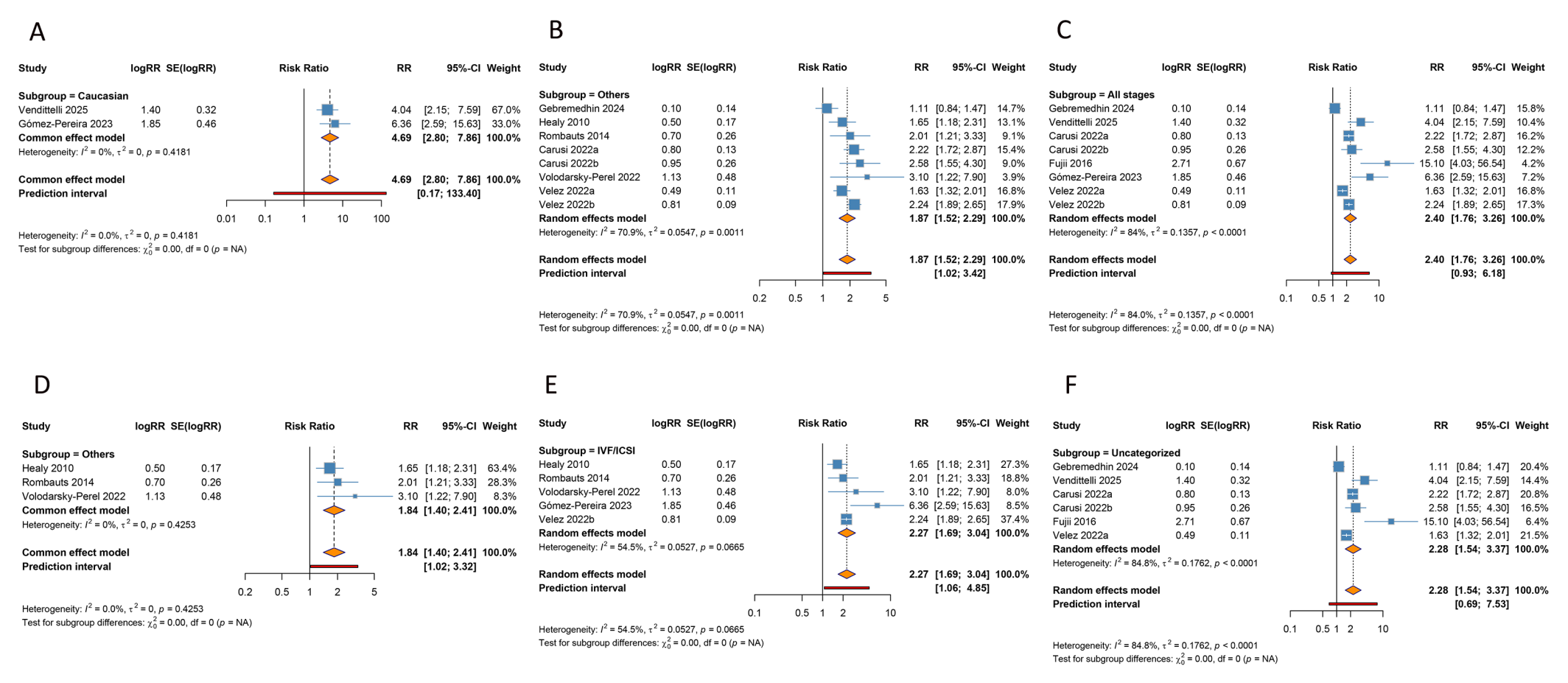


**FIGURE S6** Subgroup analysis of miscarriage. (A) Ethnicity = Caucasian; (B) Ethnicity = Asian; (C) Ethnicity = Others; (D) Endometriosis stage = All stages; (E) Endometriosis stage = Others; (F) Mode of ART = IVF/ICSI; (G) Mode of ART = Uncategorized.


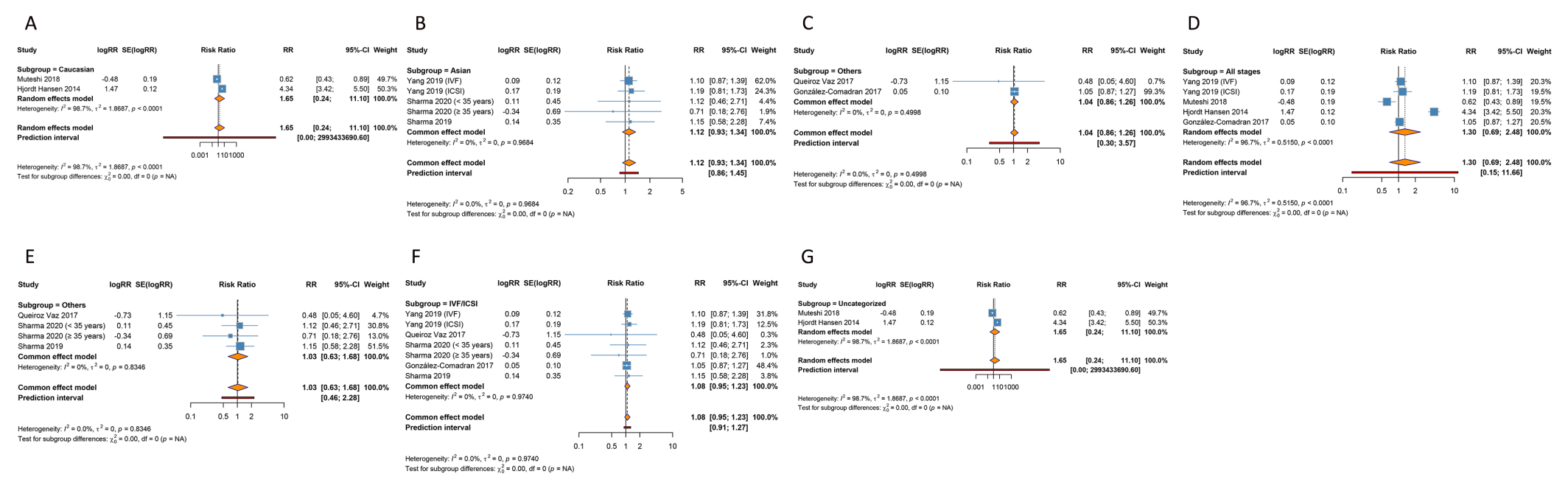


**FIGURE S7** Subgroup analysis of preeclampsia. (A) Ethnicity = Caucasian; (B) Endometriosis stage = All stages; (C) Mode of ART = Uncategorized.


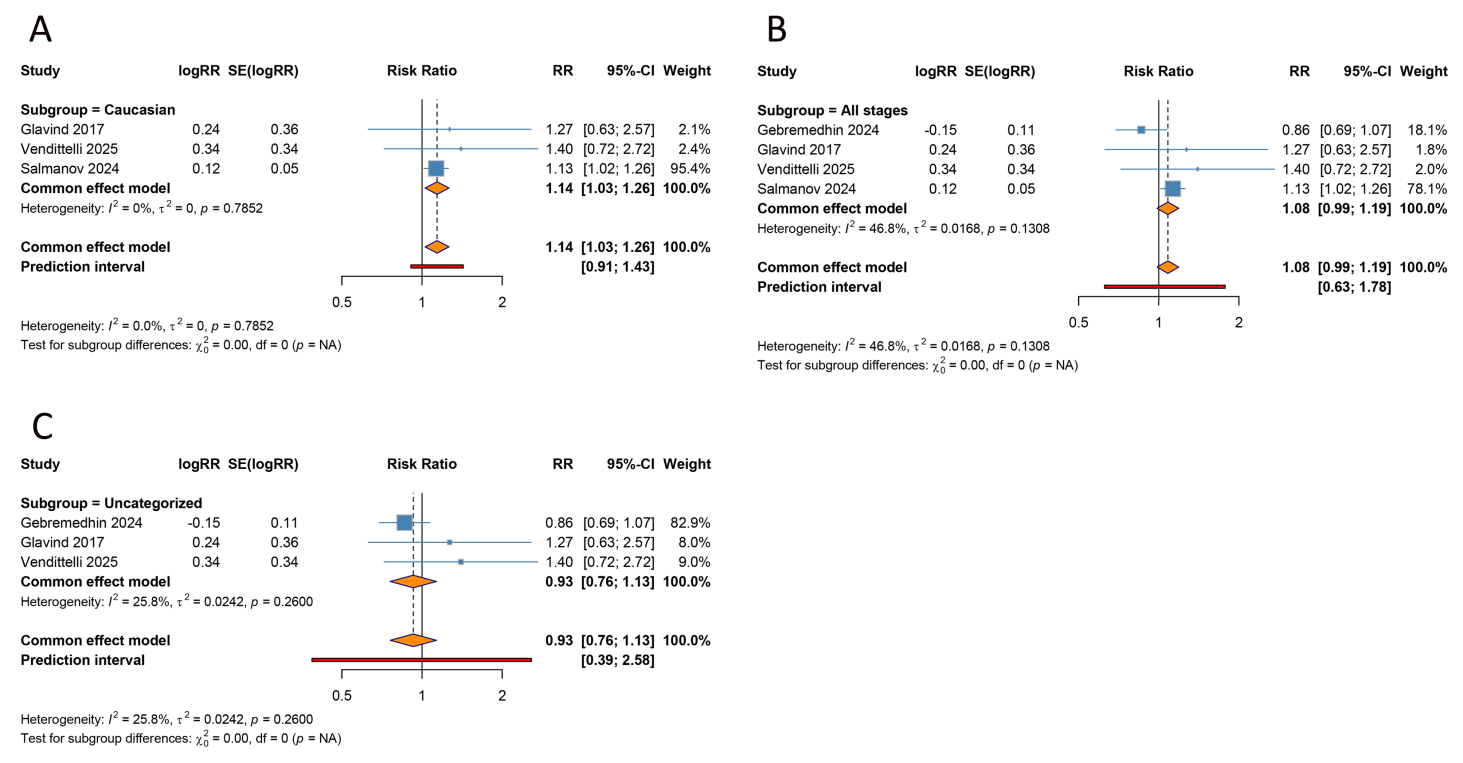


**FIGURE S8** Subgroup analysis of postpartum hemorrhage. (A) Ethnicity = Caucasian; (B) Ethnicity = Others; (C) Endometriosis stage = All stages; (D) Mode of ART = IVF/ICSI; (E) Mode of ART = Uncategorized.


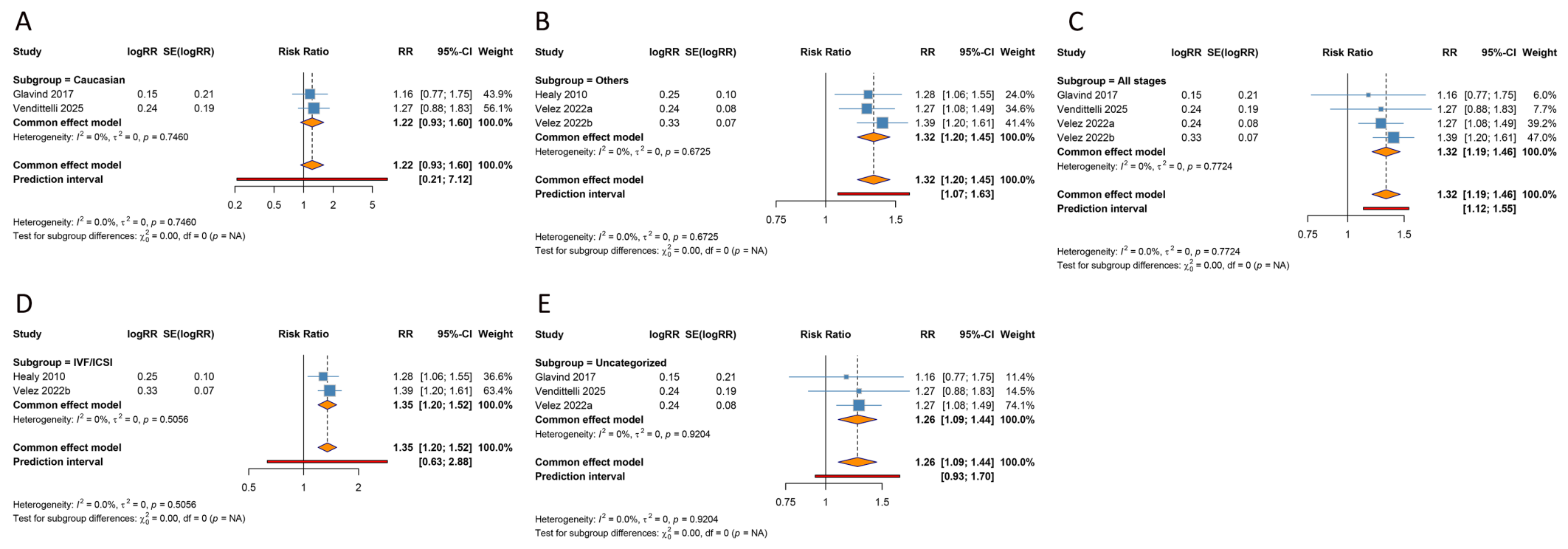


**FIGURE S9** Subgroup analysis of cesarean section. (A) Ethnicity = Caucasian; (B) Ethnicity = Others; (C) Endometriosis stage = All stages; (D) Mode of ART = IVF/ICSI; (E) Mode of ART = Uncategorized.


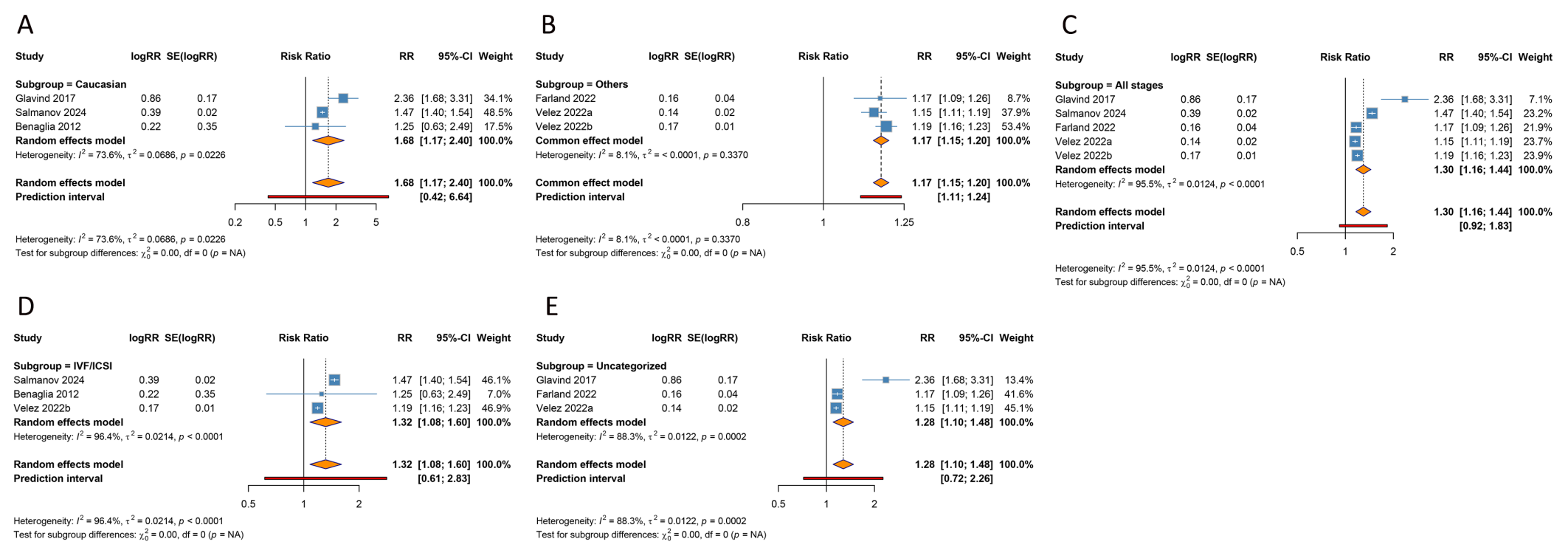


**FIGURE S10** Subgroup analysis of low birth weight. (A) Ethnicity = Caucasian; (B) Ethnicity = Asian; (C) Endometriosis stage = All stages; (D) Endometriosis stage = Others; (E) Mode of ART = IVF/ICSI.


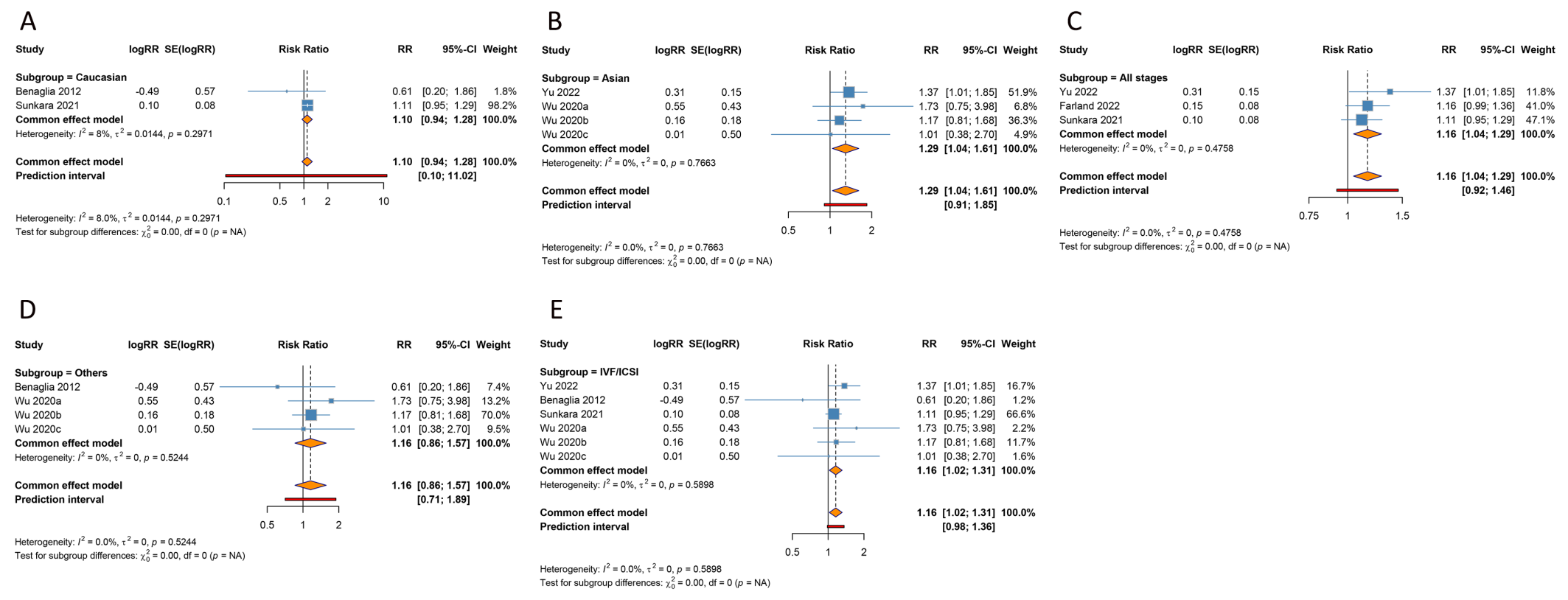


**FIGURE S11** Subgroup analysis of large for gestational age. (A) Ethnicity = Asian; (B) Ethnicity = Others; (C) Endometriosis stage = All stages; (D) Endometriosis stage = Others; (E) Mode of ART = IVF/ICSI; (F) Mode of ART = Uncategorized.


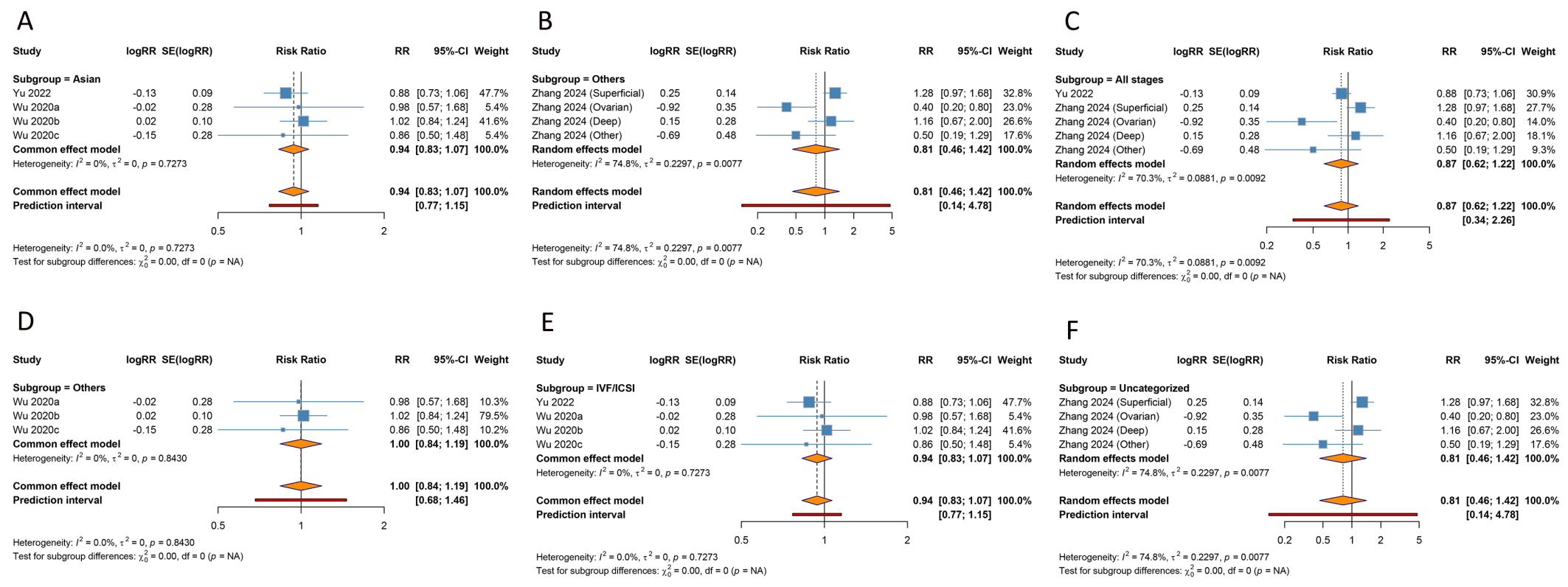


**FIGURE S12** Subgroup analysis of ectopic pregnancy. (A) Ethnicity = Caucasian; (B) Endometriosis stage = All stages; (C) Mode of ART = Uncategorized.


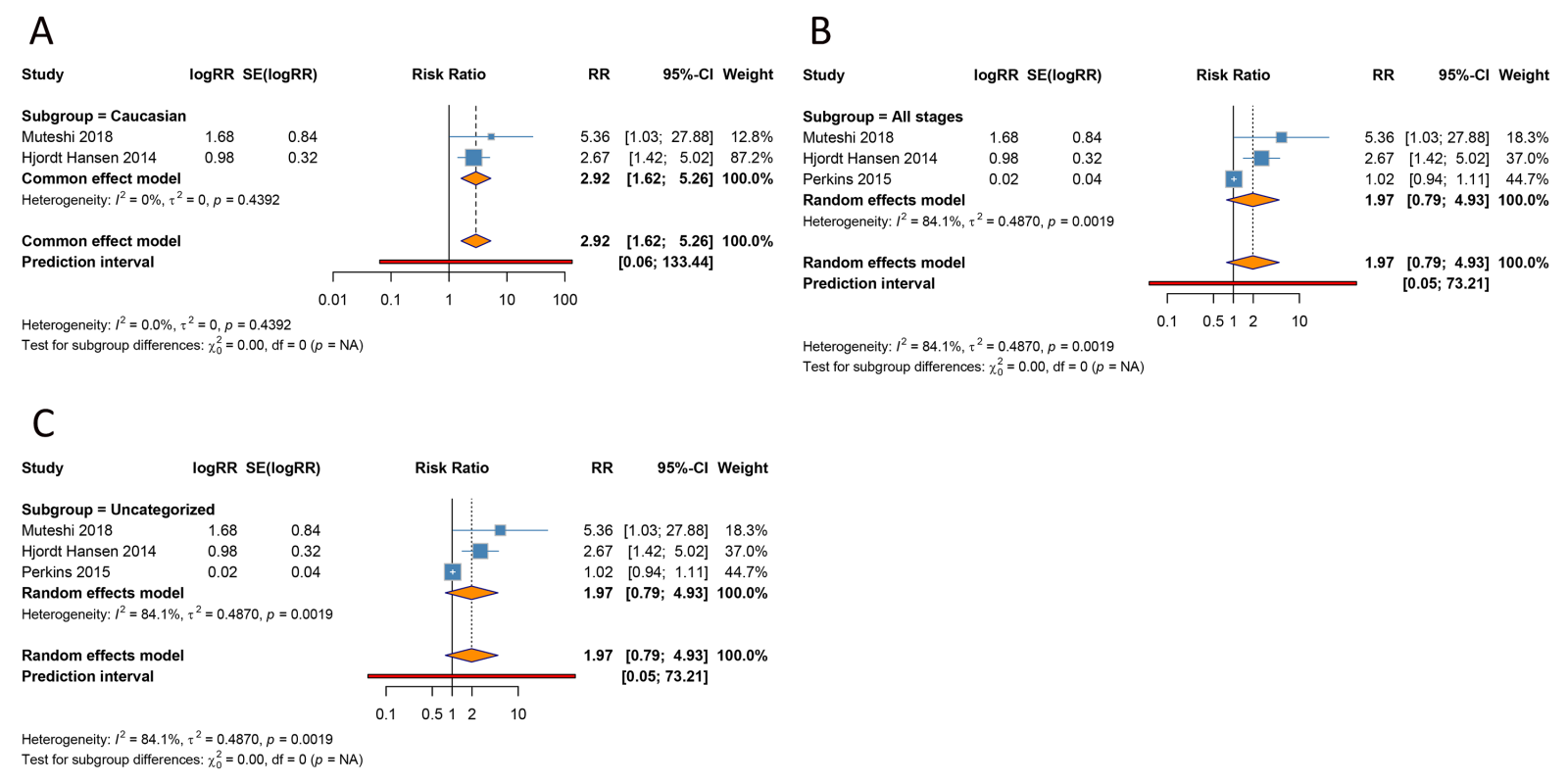


**FIGURE S13** Subgroup analysis of stillbirth. (A) Ethnicity = Caucasian; (B) Ethnicity = Others; (C) Endometriosis stage = All stages; (D) Mode of ART = IVF/ICSI; (E) Mode of ART = Uncategorized.


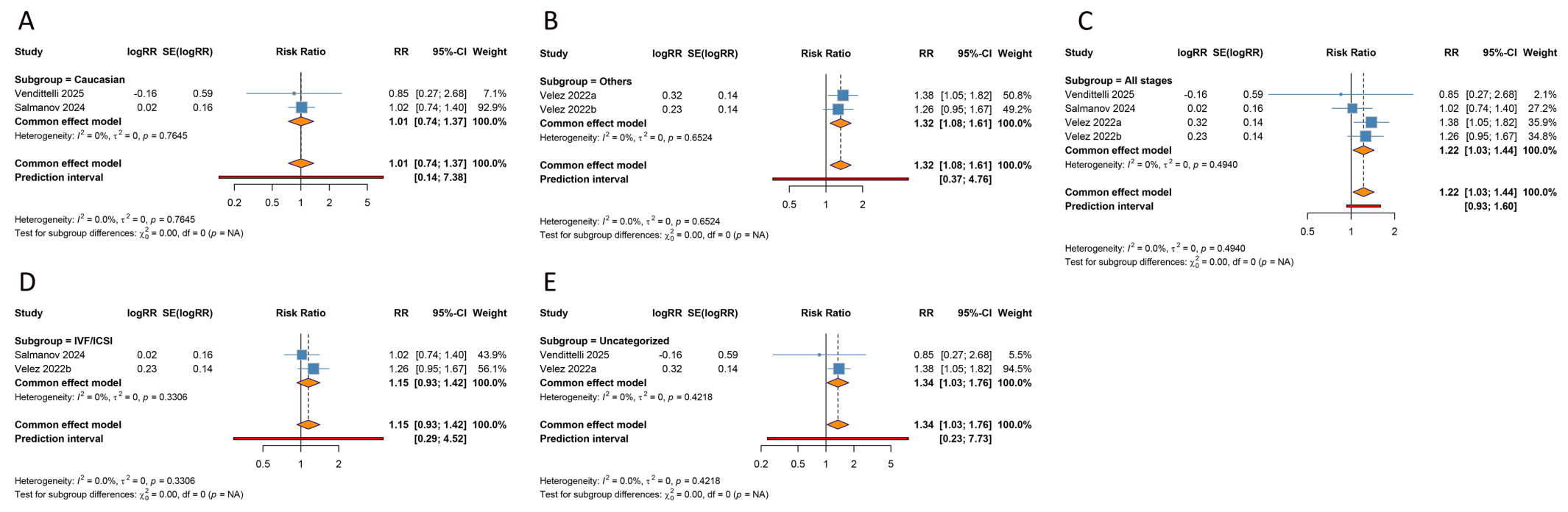


**FIGURE S14** Subgroup analysis of hypertensive disorders of pregnancy. (A) Ethnicity = Others; (B) Endometriosis stage = All stages; (C) Mode of ART = IVF/ICSI; (D) Mode of ART = Uncategorized.


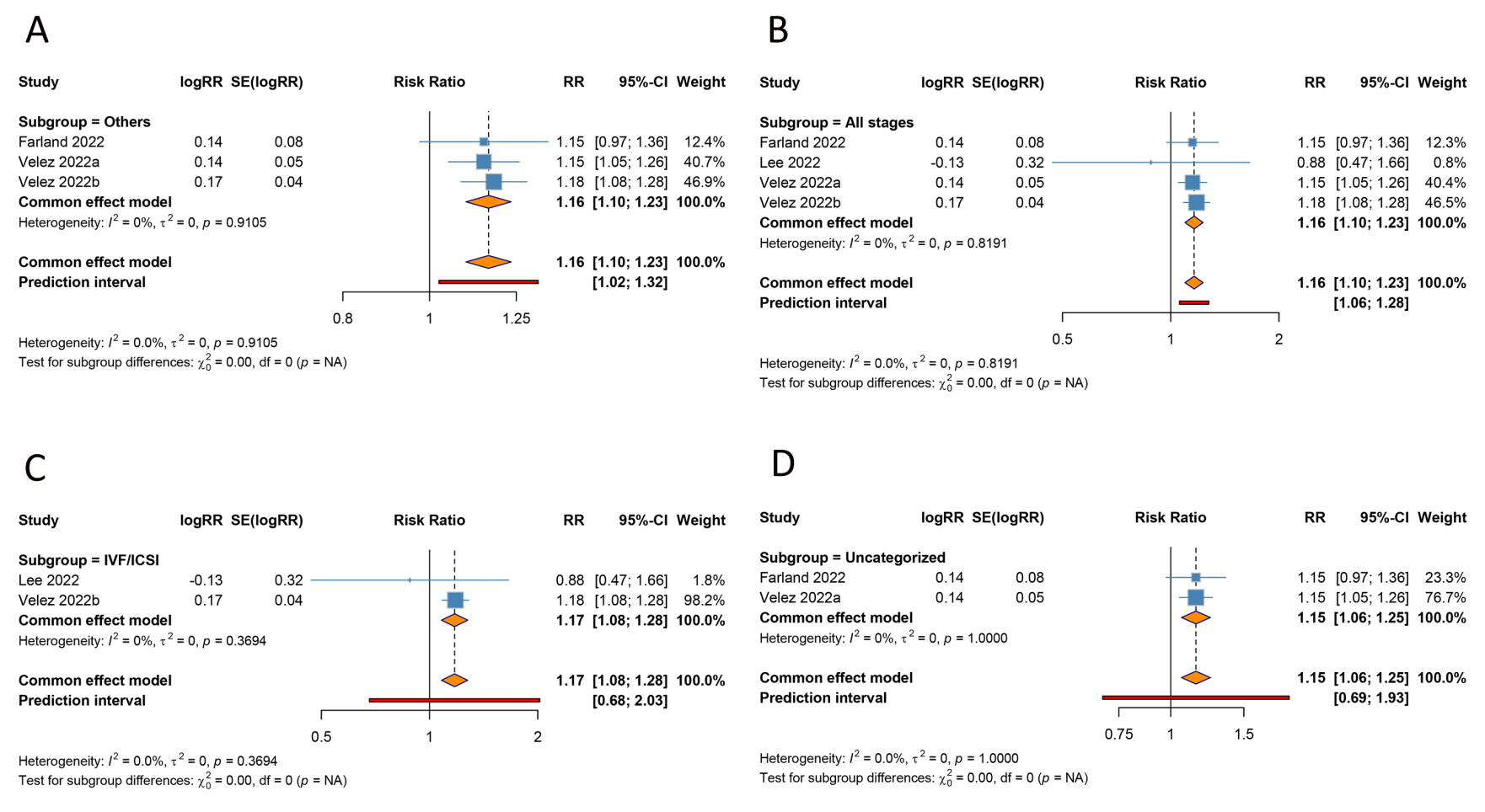


**FIGURE S15** Sensitivity analysis for the pooled results of adverse pregnancy outcomes which included ≥ 10 studies. (A) Preterm birth; (B) Small for gestational age; (C) Placenta previa.


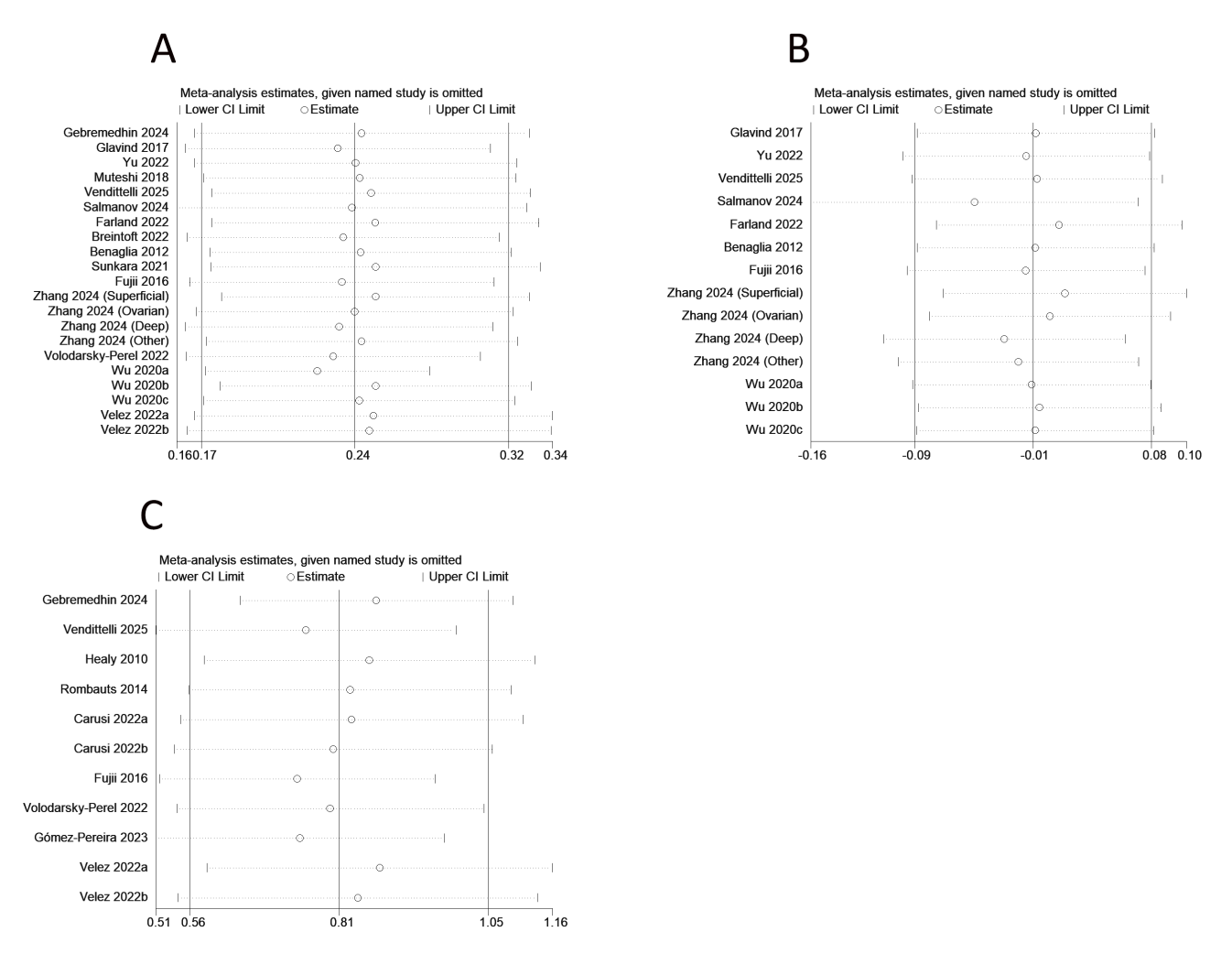


**FIGURE S16** Funnel plots of the pooled results of adverse pregnancy outcomes which included ≥ 10 studies. (A) Preterm birth; (B) Small for gestational age; (C) Placenta previa.


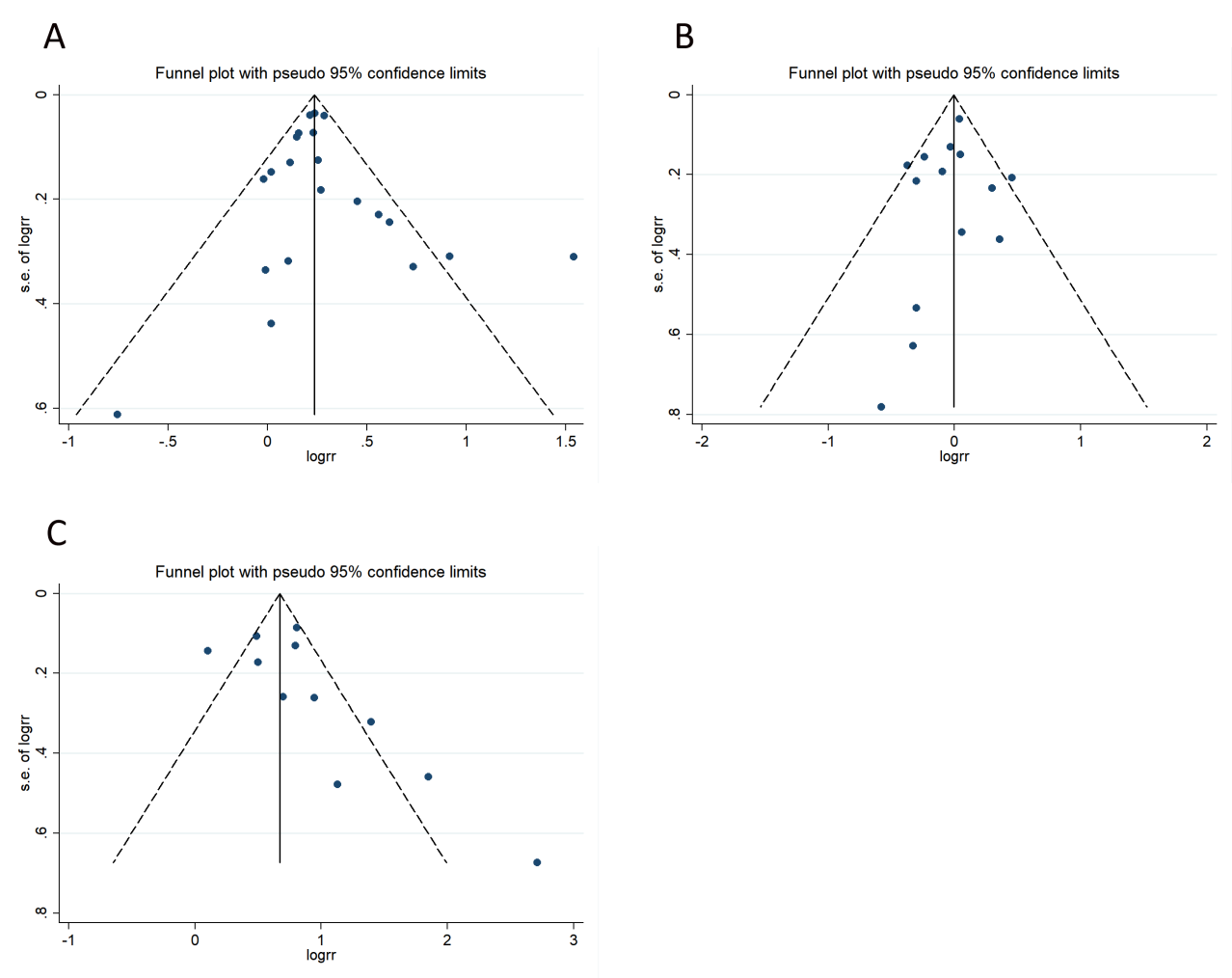

Supplement: Supplementary file 2 [file Supplementary_file_2.docx]
