# Supplementary material for: Association between endometriosis and adverse reproductive and perinatal outcomes in women undergoing assisted reproductive technology: a systematic review and meta-analysis
Source: Front Med (Lausanne). 2026 Jan 28;13:1630529. doi: 10.3389/fmed.2026.1630529 (PMC12891222; doi:10.3389/fmed.2026.1630529)
Supplement: Supplementary file 3 [file Table_1.docx]

| **TABLE S1** The Newcastle-Ottawa quality assessment scale of the included cohort studies. | | | | | | | | | | | | |
| --- | --- | --- | --- | --- | --- | --- | --- | --- | --- | --- | --- | --- |
| Study | Selection | | | |  | Comparability | |  | Assessment of outcome | | | Total score |
|  | Representativeness of exposure arm(s) | Selection of the comparative arm(s) | Origin of exposure source | Demonstration that outcome of interest was not present at start of study |  | Studies controlling the most important factors | Studies controlling the other main factors |  | Assessment of outcome with independency | Adequacy of follow-up length | Lost to follow-up acceptable |  |
| Gebremedhin (2024) | 1 | 1 | 1 | 1 |  | 1 | 0 |  | 1 | 1 | 1 | 8 |
| Yang (2019) | 1 | 1 | 1 | 1 |  | 1 | 0 |  | 1 | 1 | 1 | 8 |
| Glavind (2017) | 1 | 1 | 1 | 1 |  | 1 | 0 |  | 1 | 1 | 1 | 8 |
| Yu (2022) | 1 | 1 | 1 | 1 |  | 0 | 0 |  | 1 | 1 | 1 | 7 |
| Muteshi (2018) | 1 | 1 | 1 | 1 |  | 1 | 0 |  | 1 | 1 | 1 | 8 |
| Vendittelli (2025) | 1 | 1 | 1 | 1 |  | 1 | 0 |  | 1 | 1 | 1 | 8 |
| Salmanov (2024) | 1 | 1 | 1 | 1 |  | 0 | 0 |  | 1 | 1 | 1 | 7 |
| Healy (2010) | 1 | 1 | 1 | 1 |  | 1 | 0 |  | 1 | 1 | 1 | 8 |
| Farland (2022) | 1 | 1 | 1 | 1 |  | 1 | 0 |  | 1 | 1 | 1 | 8 |
| Breintoft (2022) | 1 | 1 | 1 | 1 |  | 1 | 0 |  | 1 | 1 | 1 | 8 |
| Queiroz Vaz (2017) | 1 | 1 | 1 | 1 |  | 0 | 0 |  | 1 | 1 | 1 | 7 |
| Benaglia 2012 | 1 | 1 | 1 | 1 |  | 1 | 0 |  | 1 | 1 | 1 | 8 |
| Sharma (2020) | 1 | 1 | 1 | 1 |  | 1 | 0 |  | 1 | 1 | 1 | 8 |
| Rombauts (2014) | 1 | 1 | 1 | 1 |  | 0 | 0 |  | 1 | 1 | 1 | 7 |
| Wu (2021) | 1 | 1 | 1 | 1 |  | 1 | 0 |  | 1 | 1 | 1 | 8 |
| Sunkara (2021) | 1 | 1 | 1 | 1 |  | 1 | 0 |  | 1 | 1 | 0 | 7 |
| Hjordt Hansen (2014) | 1 | 1 | 1 | 1 |  | 1 | 0 |  | 1 | 1 | 1 | 8 |
| Carusi (2022) | 1 | 1 | 1 | 1 |  | 1 | 0 |  | 1 | 1 | 1 | 8 |
| González-Comadran (2017) | 1 | 1 | 1 | 1 |  | 1 | 0 |  | 1 | 1 | 1 | 8 |
| Fujii (2016) | 1 | 1 | 1 | 1 |  | 1 | 0 |  | 1 | 1 | 1 | 8 |
| Sharma (2019) | 1 | 0 | 1 | 1 |  | 1 | 0 |  | 1 | 1 | 1 | 7 |
| Zhang (2024) | 1 | 1 | 1 | 1 |  | 1 | 0 |  | 1 | 1 | 1 | 8 |
| Volodarsky-Perel (2022) | 1 | 1 | 1 | 1 |  | 1 | 0 |  | 1 | 1 | 1 | 8 |
| Perkins (2015) | 1 | 1 | 1 | 1 |  | 0 | 0 |  | 1 | 1 | 1 | 7 |
| Wu (2020) | 1 | 1 | 1 | 1 |  | 1 | 0 |  | 1 | 1 | 0 | 7 |
| Alson (2024) | 1 | 1 | 1 | 1 |  | 1 | 0 |  | 1 | 1 | 1 | 8 |
| Gómez-Pereira (2023) | 1 | 1 | 1 | 1 |  | 0 | 0 |  | 1 | 1 | 1 | 7 |
| Lee (2022) | 1 | 1 | 1 | 1 |  | 0 | 0 |  | 1 | 1 | 1 | 7 |
| Velez (2022) | 1 | 1 | 1 | 1 |  | 1 | 0 |  | 1 | 1 | 1 | 8 |
